# Triple-layered nanogel for targeted and sustained delivery of florfenicol against bacterial enteritis

**DOI:** 10.1016/j.ijpx.2026.100637

**Published:** 2026-08-12

**Authors:** Tianhui Wang, Xingwang Ge, Yidan Zhang, Zhiyi Ge, Aizhi Han, Samah Attia Algharib, Muhammad Usman Ghani, Shuo Han, Xiuge Gao, Wanhe Luo

**Affiliations:** a Engineering Laboratory for Tarim Animal Diseases Diagnosis and Control, College of Animal Science and Technology, Tarim University, Alar 843300, China; b Engineering Center of Innovative Veterinary Drugs, Center for Veterinary Drug Research and Evaluation, College of Veterinary Medicine, Nanjing Agricultural University, Nanjing 210095, China; c Instrumental Analysis Center, Tarim University, Alar 843300, China; d Department of Clinical Pathology, Faculty of Veterinary Medicine, Benha University, Moshtohor, Toukh 13736, Egypt

**Keywords:** *Escherichia coli*, Triple-layered core-shell nanogel, Targeted delivery, Florfenicol, Sustained release

## Abstract

Bacterial enteritis poses a significant challenge to global public health and the livestock industry, necessitating the urgent development of an oral antibacterial formulation with high efficiency, amplified targeting capability, and sustained release. In this study, the triple-layered core-shell nanogel composed of sodium carboxymethyl cellulose, sodium tripolyphosphate, and quaternized chitosan was successfully constructed *via* a layer-by-layer self-assembly strategy for the targeted delivery and sustained release of florfenicol. The prepared triple-layered core-shell nanogel exhibited uniform spherical morphology with 110.0 ± 2.7 nm and −21.2 ± 2.6 mV, and demonstrated excellent hydrogel stability, pH-responsive release behavior, and optimal biosafety standards. Moreover, the triple-layered core-shell nanogel demonstrated elevated bactericidal potency against *Escherichia coli* relative to the free drug, and the time-kill assay together with bacterial viability assay results confirmed its concentration-dependent bactericidal effect. Scanning electron microscopy further revealed that the nanogel exerted synergistic antibacterial action by inducing bacterial membrane perturbation. *In vivo* distribution and therapeutic effect indicated that the nanogel possessed excellent retention capacity in the gastrointestinal tract, achieving satisfactory survival rate and cure rate in infected mice, while effectively limiting the excessive upregulation of pro-inflammatory cytokines (TNF-α, IL-1β and IL-6) and promoting the recovery of the anti-inflammatory cytokine (IL-10), thereby re-establishing the inflammatory and immunomodulatory balance. Collectively, the triple-layered core-shell nanogel developed in this study provides an efficient and safe nanoplatform for the oral treatment of bacterial enteritis through a triple synergistic mechanism involving targeted delivery, enhanced antibacterial activity and immunomodulation.

## Graphical abstract

**Figure:**
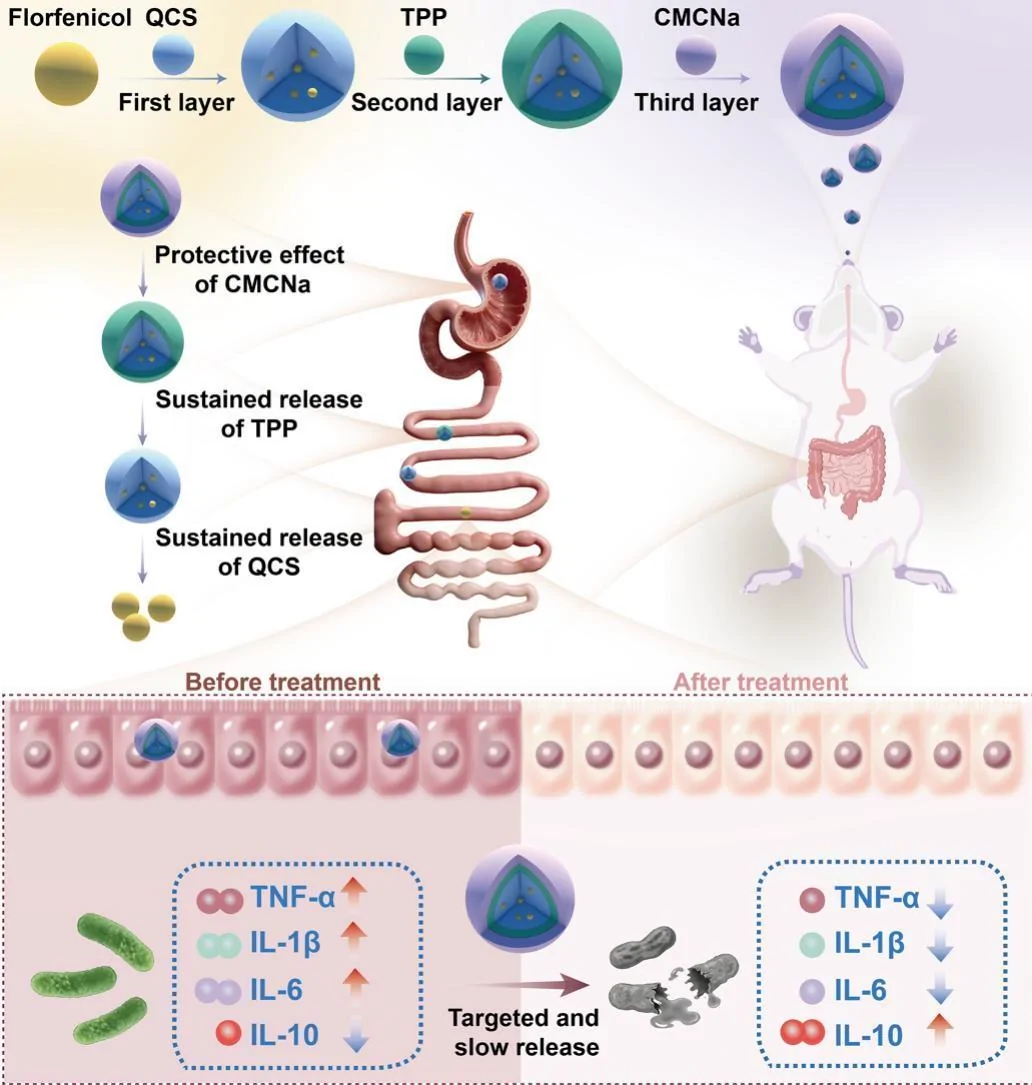

## 1 Introduction

Enteritis infection is recognized as a principal determinant of global clinical burden and fatalities (Zhang et al., 2024a; Sands et al., 2021; Lin et al., 2025). Notably, enteritis induced by pathogenic *Escherichia coli* (particularly enteropathogenic and enterotoxigenic *E. coli*, *etc.*) indicates a pressing concern for both public and animal health (Ma et al., 2025). In regions with poor sanitation, *E. coli* is identified as a primary etiological agent of bacterial diarrhea, leading to severe dehydration, electrolyte imbalance, and growth retardation (Jiao et al., 2024). Within the livestock industry, especially in intensive pig, poultry, and calf farming, *E. coli*-associated enteritis (such as yellow scours and white scours in piglets, and avian colibacillosis) results in substantial economic losses, which are manifested by high morbidity and mortality rates, reduced productivity, and significant costs associated with treatment and prevention (Arbab et al., 2022; Muleme et al., 2023; Li et al., 2024). More critically, certain strains, like Shiga toxin-producing *E. coli* (STEC), can trigger fatal complications,including hemolytic uremic disorder. Bacterial enteritis, characterized by the adhesion, colonization, invasion, or toxin production of *E. coli* on the intestinal mucosa, leads to the disruption of the intestinal barrier function and provokes severe inflammatory diarrhea. This process can causelocal tissue damage, and the intense inflammatory cascade may also compromise intestinal immune homeostasis, impair nutrient absorption, and, in severe cases, facilitate bacterial translocation, potentially resulting in mortal consequences in the form of bacteremia (Gökmen et al., 2024; Askari Badouei et al., 2023; Chevarin et al., 2024). Therefore, effective suppression of *E. coli* is crucial for the combating bacterial enteritis. Florfenicol, a broad-spectrum amphenicol antibiotic, is employed as one of the therapeutic agents against *E. coli* infections in veterinary practice due to its Broad-spectrum antibacterial activity targeting Gram-negative and Gram-positive bacteria, including *E. coli (*Kerek et al., *2023**;* Zhang et al., *2024b**;* Wang et al., *2025**)*. Extensive *in vitro* susceptibility data indicate that the majority of clinically isolated pathogenic *E. coli* strains are highly susceptible to florfenicol with minimum inhibitory concentration (MIC) (*e.g.*, MIC values for many strains are between 2 and 8 μg/mL) (Luo et al., 2024; Luo et al., 2025a). However, the clinical application of florfenicol in veterinary medicine is severely limited by its inherent lack of targeting ability and controlled-release properties (Wang et al., 2016; Luo et al., 2025b).

Consequently, nano drug delivery system have garnered increasing interest for their potential to offer: 1) oral stability and resistance to gastric degradation; 2) controllable drug release in response to the intestinal microenvironment (*e.g.*, pH, enzymes, redox potential) or *via* programmed temporal release; and 3) enhanced antibacterial effects, potentially through the intrinsic antimicrobial activity of the carrier materials (Ding et al., 2022; Wang et al., 2022). Among these, nanogel delivery systems, defined as Physically or chemically cross-linked three-dimensional networks of hydrophilic/amphiphilic polymers exhibit distinctive advantages (Chen et al., 2018). Their highly hydratable configuration facilitates the robust loading of both hydrophilic and hydrophobic drugs (Zhang et al., 2023). Nanogel offer high drug-loading capacity, remarkable biocompatibility, tunable size and surface properties, and the ability to undergo responsive swelling/deswelling to external stimuli (*e.g.*, pH), facilitating intelligent drug release, which is beneficial for targeted therapy of bacterial enteritis (Zhu et al., 2023). The successful construction of nanogel for treating *E. coli* enteritis hinges on the judicious selection of biomaterials and sophisticated molecular design (Li et al., 2021). Sodium carboxymethyl cellulose (CMCNa), an anionic cellulose derivative, possesses favorable attributes such as good aqueous solubility, film-forming capacity, biocompatibility, and biodegradability. It also demonstrates gastric acid tolerance, protecting encapsulated drugs from breakdown in the proximal gastrointestinal tract and thereby maintaining oral delivery stability (Pagano et al., 2021; Sharratt et al., 2021). Furthermore, its inherent viscosity can act as a physical barrier to modulate drug release kinetics. In addition, chitosan (CS), a natural cationic polysaccharide, is highly regarded for its biocompatibility, biodegradability, mucoadhesive properties, and intrinsic antimicrobial activity. It can adsorb onto the anionic bacterial cell membrane *via* electrostatic forces, disrupting membrane integrity and causing intracellular content leakage (Mohammadi et al., 2021; Hu and Luo, 2021). However, the major limitation of CS is its insufficient hydrophilicity, being soluble only in acidic media, which severely restricts its processing and application under physiological conditions. To circumvent this challenge, chemical modification by introducing quaternary ammonium groups is performed, yielding quaternized chitosan (QCS) (Liang et al., 2021). QCS exhibits excellent solubility across a broad pH range (from acidic to alkaline), which significantly expands its applicability (Sun et al., 2021).

**Fig. 1:**
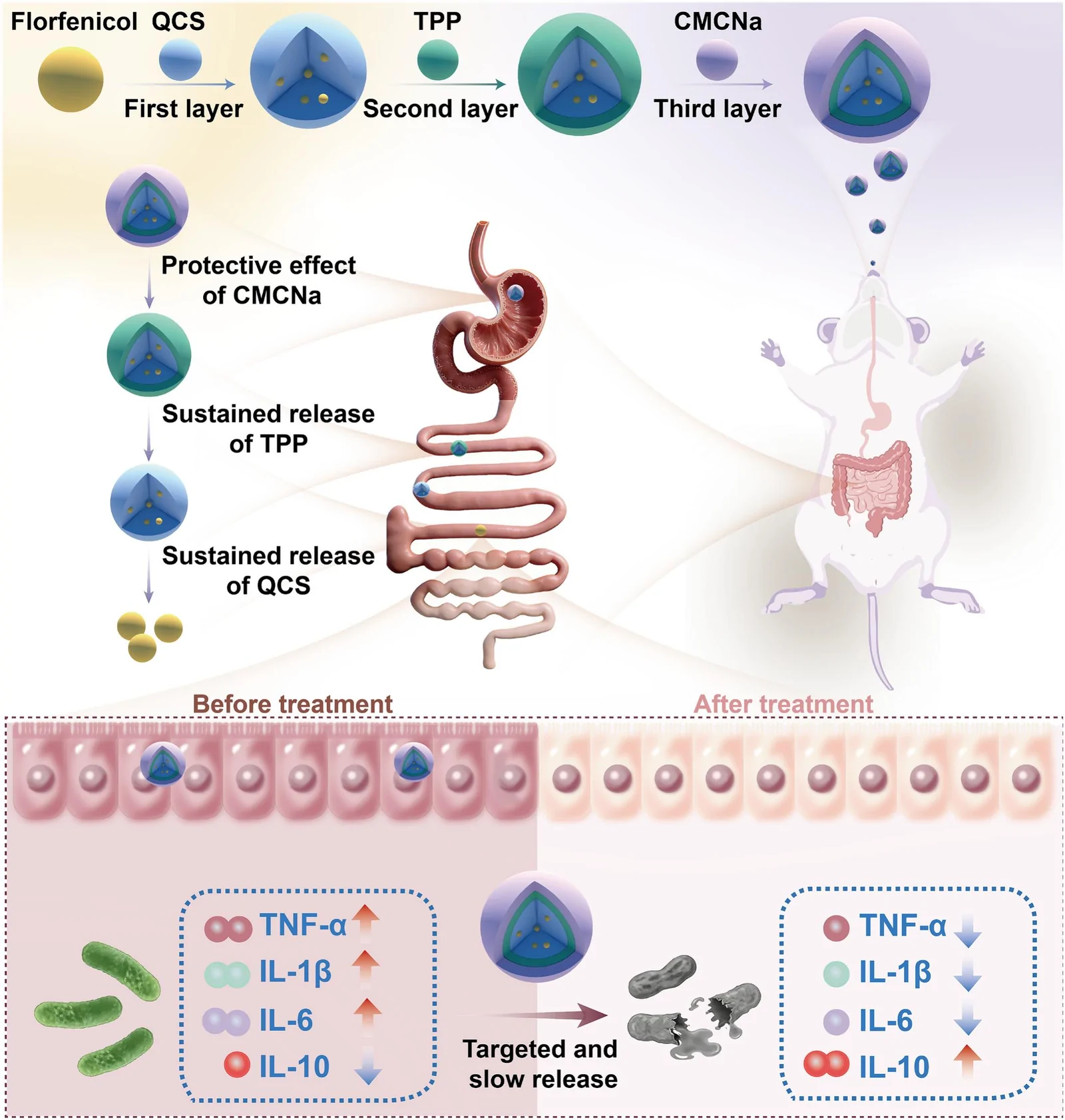
Schematic diagram of the triple-layered core-shell nanogel as an efficient and safe nanoplatform for the oral treatment of bacterial enteritis through a triple synergistic mechanism involving targeted delivery, enhanced antibacterial activity, and immunomodulation.

Considering this, a novel triple-layered core-shell nanogel was designed in this study, aiming to achieve efficient, targeted, and sustained-release therapy for *E. coli* enteritis using florfenicol *via* a precise “layer-by-layer” assembly strategy. Initially, a positively charged drug-polymer composite core was formed, where hydrophobic florfenicol was encapsulated by water-soluble QCS through ionic interactions, endowing the core with both preliminary antibacterial activity and mucoadhesive potential. Subsequently, the multivalent anion tripolyphosphate (TPP) was introduced, serving a dual role: as an ionic crosslinker to enhance the compactness and stability of the core structure, and as an electrostatic bridge for the subsequent assembly of the outer layer. Finally, anionic CMCNa was electrostatically adsorbed onto the surface, completing the hierarchical nanostructure (Fig. 1). This results in a well-defined architecture comprising a drug-loaded core, an intermediate ionic cross-linked/bridging layer, and an outer functional shell. The outer CMCNa shell was expected to provide biocompatible protection and modulation of surface charge to minimize non-specific caoting, and potential for responsive degradation in the intestinal milieu, gradually exposing the underlying strongly cationic QCS. The exposed QCS could then actively target and accumulate at negatively charged infection sites, enhancing mucus penetration and bacterial membrane binding. Regarding drug release behavior, the cross-linked network established by TPP, in conjunction with the CMCNa shell, forms a diffusion barrier, enabling sustained and controlled drug release. More importantly, a sequential synergistic effect was anticipated: QCS primarily disrupts the bacterial membrane structure, after which the released florfenicol can more readily enter the bacterial cells to exert its inhibitory effect. This “membrane disruption followed by intracellular inhibition” mode of action may potentiate therapeutic efficacy while potentially reducing the required dosage and the risk of antimicrobial resistance. In summary, the triple-layered core-shell nanogel developed herein was anticipated to overcome the limitations associated with conventional florfenicol formulation. By integrating strategies for targeted delivery, responsive release, and synergistic antibacterial effect, it offers a promising, efficient, and safe therapeutic approach for *E. coli* enteritis. This work might also provide a valuable reference for the development of nanomedicine based platforms targeting other infectious intestinal diseases.

## 2 Materials and methods

### 2.1 Materials

Florfenicol active pharmaceutical ingredients (API) (98.0%, MV: 358.21), CMCNa (Viscosity: 600–1000 mpa.s), TPP (98.0%; MV: 367.86) and CS (Deacetylation degree ≥90%, MW: 100000) were purchased from Macklin (China). Dingyuan Co., Ltd. (China) provided the phosphate-buffered saline (PBS), 3-chloro-2-hydroxypropyltrimethylammonium chloride (CTA), 1,1’-Dioctadecyl-3,3,3′,3′-tetramethylindotricarbocyanine iodide (DiR), tumor necrosis factor-alpha (TNF-α), interleukin-1β (IL-1β), interleukin-6 (IL-6) and interleukin-10 (IL-10) quantitative assay kit, Mueller-Hinton broth and tryptic soy broth (TSB). A Live/Dead backlit bacterial viability kit was obtained from Shanghai Bestbio Biotechnology Co., Ltd. (China). An *E. coli* isolate and *S. aureus* isolate were provided by the Tarim University Engineering Laboratory for Tarim Animal Diseases Diagnosis and Control (China). A Milli-Q system was used to prepare the ultrapure water (Millipore, USA). The remaining reagents in the text were of either equivalent or analytical grade.

### 2.2 Synthesis of QCS

The nucleophilic substitution reaction employing CTA as the quaternizing agent was conducted to synthesize quaternized chitosan, thereby enhancing its water solubility and augmenting its cationic charge. Briefly, an accurate mass of 2.0 g CS was dispersed in 50 mL of isopropanol. The mixture was agitated with magnetic stirrer at room temperature for 30 min to facilitate and swelling of the CS. Subsequently, 5.0 g NaOH was dissolved in 15 mL of ultrapure water to prepare alkaline solution and added dropwise to the dispersion. The alkalization process was subjected to stirring at ambient temperature for 2 h to activate the amino reactive sites on the chitosan molecular chains. For the quaternization reaction, 9.3 g CTA (the molar ratio of CTA: CS = 4:1) was solubilized in 10 mL ultrapure water and titrated into the reaction system. The temperature was then raised to 80 °C, and the process was maintained under constant stirring for 12 h. The reaction mixture was then cooled to room temperature and the pH precisely adjusted to 7.0 with a dilute hydrochloric acid in order to end the reaction. The resulting solution was poured into five volumes of anhydrous ethanol, and the product was precipitated with vigorous stirring. After filtration under reduced pressure, the solid residue was thoroughly rinsed three times successively using anhydrous ethanol and acetone to completely remove unreacted small molecules and the byproduct. Finally, the solid product was dried in vacuum at 50 °C for 24 h and produced QCS with much greater aqueous solubility. Fourier transform infrared (FTIR) spectroscopy (Nicolet iS50, Thermo Scientific Inc., USA) was used to determine the chemical structure of the synthesized QCS.

### 2.3 Formulation of triple-layered core-shell nanogel

The triple-layered core-shell nanogel were prepared by electrostatic interactions. Briefly, 20 and 10 mL of deionized water were employed to solubilize CMCNa (0.6, 1.2 or 1.8 g) and TPP (0.1, 0.2 or 0.4 g). Florfenicol (0.5 g) and QCS (0.1, 0.5 or 1.0 g) were dissolved in 10 mL dimethyl formamide and 10 mL PBS, correspondingly. Thereafter, the florfenicol solution was introduced dropwise into the QCS solution under magnetic stirring at 1000 rpm in order to generate the primary-layer encapsulate nanoparticles. The resulting primary-layer nanoparticles were then introduced dropwise into the TPP solution to obtain the secondary-layer encapsulated nanoparticles. Finally, the secondary-layer encapsulated nanoparticles were gradually added into the CMCNa solution to fabricate the tertiary-layer encapsulated nanoparticles, namely triple-layered core-shell nanogel. The ideal concentrations were determined using the loading capacity (LC) and the encapsulation efficiency (EE) of QCS, TPP and CMCNa.

### 2.4 Box-Behnken response surface analysis

The best concentrations of QCS, TPP, and CMCNa were accurately calculated with the help of the Design-Expert 8.0 software using EE and LC as the evaluation indices. The factors and levels of the Box-Behnken design were showed in Table S1.

### 2.5 Characterization

#### 2.5.1 Morphological observation

The morphology, lyophilization weight and re-dispersibility of the triple-layered core-shell nanogel were analyzed. The transmission electron microscopy (TEM, JEM-2100Plus, Japan) and scanning electron microscopy (SEM, APREOS, USA) were used to perform morphological characterization. Prior to TEM analysis, the nanogels were diluted 100-fold and dispersed by ultrasonic cleaning for 30 min using an ultrasonicator (BILON3-120A, China). The sample was then placed on copper grids with thin films, negatively stained with 2% sodium phosphotungstate, and equilibrated to dryness at room temperature before imaging. For SEM observation, lyophilized nanogel placed on silicon wafers were deposited with thin layer of gold *via* ion sputtering and analyzed at an applied voltage of 20 kV after oven drying.

#### 2.5.2 The mean size, zeta potential (ZP), and polydispersity index (PDI)

The mean size, ZP, and PDI of the first layer nanoparticles, the secondary layer nanoparticles and the triple-layered core-shell nanogel were determined using a Zetasizer ZX3600 (Malvern Panalytical, UK). Prior to measurement, the samples were serially diluted 100-fold and placed in a sonication bath for 30 min. Each respective sample was analyzed thrice, and the results were presented as mean ± standard deviation.

#### 2.5.3 FTIR

The first layer nanoparticles, the secondary layer nanoparticles and the triple-layered core-shell nanogel were first lyophilized (FDU-1200, Japan). Subsequently, FTIR spectroscopy was employed to investigate the interactions among QCS, TPP, and CMCNa within the formulation. Briefly, the lyophilized powder was finely ground and compressed into a disc. FTIR spectra were then recorded 32 infterograms by scanning the discat a resolution of 4 cm^−1^ and a velocity of 2 mm/s, using carson apodization.

#### 2.5.4 Rheological analysis

Rheological properties of the triple-layered core-shell nanogel was characterized using a HAAKE MARS RS6000 rheometer (Germany) equipped with a parallel plate (P20 TiL). Oscillatory Measurements were performed at 37 °C with the following parameters set at: stress of 1.0 Pa, frequency of 1.0 Hz, gap of 1.000 mm, sample volume of 0.4 mL, and duration of 30.0 min. Specifically, a time dependent analysis was conducted to evaluate the storage modulus (G') and loss modulus (G") of the triple-layered shell-core nanogel.

#### 2.5.5 Responsive performances

To evaluate pH-responsive behavior, the triple-layered core-shell nanogel was first incubated in SGF and SIF for 24 h. Subsequently, the samples were collected at set intervals (0.5, 1, 2, 4, 6, 8, 12 and 24 h), and the concentrations were assessed. Meanwhile, the degradation behavior was observed at different time points. Finally, the cumulative release profiles of the triple-layered core-shell nanogel was established.

### 2.6 Stability

The stability of triple-layered core-shell nanogel was evaluated through influencing-factor studies under elevated temperature (40 °C), high humidity (90 ± 5% RH), and intense light (4500 ± 500 lx) for 10 days. The samples were collected on days 5 and 10 to monitor morphological changes, mean size, ZP, PDI, as well as pharmaceutical parameters (LC and EE) and spectral properties (FTIR).

### 2.7 Antibacterial activity

#### 2.7.1 Determination of the MIC

The MIC of florfenicol API and the triple-layered core-shell nanogel against the *E. coli* isolate and *S. aureus* isolate were evaluated using the macrodilution assay. Specifically, both florfenicol formulations were prepared in Mueller-Hinton broth across graded concentrations (128, 64, 32, 16, 8, 4, 2, 1, 0.5, 0.25, 0.125, 0.0625, and 0.03125 μg/mL). Meanwhile, the bacterial inoculcum was adjusted to a target concentration of 1 × 10^6^ CFU/mL. Following incubation for 24 h, the MIC was defined as the lowest effective concentration of the drug that demonstrate antibacterial activity. All tests were executed in triplicate to achieve reproducibility.

#### 2.7.2 Inhibition zones

The antibacterial efficacy of florfenicol API and the triple-layered core-shell nanogel was assessed by measuring their inhibition zones against *E. coli* isolate and *S. aureus* isolate. To perform the assay, 15 mL of agar-based medium was poured into a sterile agar plate and allowed to solidify. Subsequently, 5 mL of agar medium inoculated with 0.1 mL of bacterial suspension (containing 1 × 10^6^ CFU/mL of *E. coli* isolate or *S. aureus* isolate) was overlaid onto the initial layer. Once the agar had solidified, three wells were punched using a sterile cork borer. Into each well, 50 μL of either florfenicol API or the triple-layered core-shell nanogel (both containing 100 μg/mL florfenicol) was added, while PBS served as the control. The agar plates were then allowed to incubateat 37 °C for 24 h under 5% CO_2_, afterwards inhibition zone diameter were measured and logged.

#### 2.7.3 Time-killing curves

The *in vitro* killing kinetics of the triple-layered encapsulated nanogel against the *E. coli* isolate and *S. aureus* isolate was assessed by generating time-kill curves based on MICs values. Specifically, the triple-layered core-shell nanogel were prepared at concentrations equivalent to 0×, 1/2×, 1×, and 2 × MIC. These drug concentrations were then combined with a bacterial suspension of 1 × 10^6^ CFU/mL. Bacterial viability was monitored over time using a real-time microbial growth monitoring system (MicroScreen-HT, China) at designated times (0, 1, 2, 4, 8, 12, and 24 h). The resulting data were used to construct time-kill curves, reflecting the dynamic changes in bacterial counts. All experiments were performed in three independent replicates, and results were summarized as mean ± standard deviation.

#### 2.7.4 Live/dead bacterial staining analysis

The bacterial suspension of *E. coli* isolate (1 × 10^6^ CFU/mL) was incubated in presence of florfenicol API and the triple-layered core-shell nanogel (both at a concentration of 1 × MIC). After two hours of exposure, the samples were treated with a live/dead fluorescent viability stain. Finally, 5 μL of the stained suspension was mounted on a glass slide and analyzed under a fluorescence microscope (TS2R-FL, Japan) to assess bacterial viability.

#### 2.7.5 Colony counting

Furthermore, the antibacterial effects were evaluated by incorporating florfenicol API and the triple-layered core-shell nanogel (both at 1 × MIC) into the culture medium. Initially, 15 mL of agar medium supplemented with 0.1 mL of each test substance was poured into sterile plates and allowed to solidify. The surface was then evenly inoculated with 0.1 mL of bacterial suspension enriched with 1 × 10^6^ CFU/mL of *E. coli* isolate or *S. aureus* isolate. PBS served as a negative control. The plates were incubated at 37 °C for 24 h under 5% CO_2_, after which bacterial growth was observed and recorded.

#### 2.7.6 Morphological analysis

In this investigation, the morphological changes in *E. coli* isolate and *S. aureus* isolate following exposure to florfenicol API and the triple-layered core-shell nanogel were examined using SEM, with PBS serving as the control. Briefly, bacterial suspensions (1 × 10^6^ CFU/mL) were incubated on cover glasses in triplicate at 37 °C for 24 h under each treatment condition at 1 × MIC. After incubation, the *E. coli* were fixed and dehydrated for SEM observation. Specifically, samples were chemically fixed with fixated solution of 2.5% glutaraldehyde at 4 °C for 2 h, then rinsed twice with PBS (15 min each). Dehydration was performed using a graded ethanol concentrations (30%, 50%, 70%, 90%, 95%, and 100%) for 20 min per step. Subsequent to critical-point drying and gold sputter-coating, all specimens were visualized under SEM.

### 2.8 Visualization study

To asses the *in vivo* distribution and drug retention profile, healthy mice were fasted for 12 h and then orally administered with DiR-labeled florfenicol API or the triple-layered core-shell nanogel at equivalent fluorescence intensities. PBS was orally administered as a control. To investigate the absorption and distribution characteristics of the nanoformulation in various organs and the gastrointestinal tract, as well as its sustained-release effect, the mice in each group were euthanized at 4 or 8 h. Vital organs (heart, liver, spleen, lungs, kidneys, and the entire gastrointestinal tract) were then excised, and fluorescence images of these isolated organs were captured.

### 2.9 Therapeutic experiments

All animal experiments were conducted in compliance with the ARRIVE guidelines and approved by the Institutional Animal Care and Use Committee of Tarim University (Approval No. 2023008). Male Kunming mice (6–8 weeks old, weighing 32–34 g at the start of the experiment) were obtained from Wuhan Pinuofei Biological Co., Ltd. (China). Mice were housed under standard conditions (12 h light/dark cycle, 22 ± 2 °C, 55 ± 5% humidity) with free access to food and water. For the therapeutic efficacy study, 24 mice were randomly assigned to four groups (*n* = 6 per group) using a computer-generated randomization scheme. No blinding was applied during the experiments. Sample sizes were selected based on preliminary experiments and previous studies to ensure adequate statistical power, with no animals excluded from the analysis. All efforts were made to minimize animal suffering in accordance with the university's Animal Science Academy Ethics Committee guidelines. The negative control group received physiological saline daily for 10 consecutive days. The positive control group was infected with *E. coli* isolate over 3 successive days and then left untreated. The two treatment groups were similarly infected for 3 days, followed by administration of either florfenicol API or the triple-layered core-shell nanogel from days 4 to 24 (21-day treatment). On day 25, therapeutic efficacy were further assessed by monitoring body weight changes, and calculating the survival rate and cure rate for each group. On the other hand, all mice were euthanized, intestinal tissues from each group were collected for hematoxylin and eosin (H&E) staining and measuring serum levels of TNF-α, IL-1β, IL-6 and IL-10 using quantitative detection kit.

### 2.10 *In vitro* and *in vivo* biosafety studies

The hemocompatibility of the triple-layered core-shell nanogel was evaluated using a red blood cell (RBC) hemolysis assay. Briefly, a 2% RBC suspension was incubated in presence of the nanogel samples under gentle shaking at 37 °C for 2 h. Physiological saline and ultrapure water were used as negative and positive controls, respectively. After incubation, the mixtures were centrifuged, and the absorbance of the supernatants was quantified at 540 nm to quantify hemoglobin release. The degree of hemolysis was then calculated based on the obtained absorbance values.

To assess the long-term safety of the triple-layered core-shell nanogel, a 14-day oral toxicity study was conducted in mice. The animals received a daily dosage of 10 mg/g of the nanogel *via* oral gavage. On day 14, all mice were euthanized, and the vital organs (heart, liver, spleen, lungs, and kidneys) were surgically excised and weighed to calculate the organ coefficients (OCs). For histopathological examination, these tissues were initially fixed in 4% formaldehyde, then subjected to dehydration through a graded ethanol series, and finally embedded in paraffin wax. Subsequently, the tissue blocks were sectioned, deparaffinized, rehydrated, and stained with H&E for microscopic analysis. Untreated mice served as the control group throughout the experiment. A comprehensive assessment of *in vivo* toxicity was performed. Histological examination of major organs was conducted *via* fluorescence microscopy (PANNORAMIC SCAN, 3DHISTECH). Blood samples were collected on day 14 of consecutive administration for physiological and biochemical analyses. The comprehensive hematological parameters measured included white blood cell count (WBC), lymphocyte count (Lym), monocyte count (Mon), neutrophil count (Neu), red blood cell count (RBC), hemoglobin (HGB), hematocrit (HCT), mean corpuscular volume (MCV), mean corpuscular hemoglobin (MCH), mean corpuscular hemoglobin concentration (MCHC), red blood cell distribution width (RDW), platelet count (PLT), mean platelet volume (MPV), platelet distribution width (PDW), and plateletcrit (PCT). The biochemical parameters assessed included total protein (TP), albumin (ALB), glucose (GLU), alanine aminotransferase (ALT), aspartate aminotransferase (AST), triglycerides (TG), creatinine (CREA), and urea (UREA). Furthermore, systemic inflammatory response was assessed by measuring serum levels of TNF-α, IL-1β, L-6 and IL-10 on day 14 using quantitative detection kit.

### 2.11 Statistical analysis

Statistical analyses were conducted *via* SPSS software (version 19.0). All experimental data are expressed as the mean ± standard deviation (SD). Comparisons among groups were analyzed employing one-way analysis of variance (ANOVA), and differences were considered statistically significant at *p* < 0.05.

## 3 Results and discussion

### 3.1 FTIR analysis of QCS

FTIR was used to characterize the chemical structural changes of CS and QCS (Fig. 3A). FTIR spectrum of CS showed a broad absorption band at 3412 cm^−1^ which was ascribed to the overlapping O—H and N—H stretching vibrations. The C—H stretching vibrations of -CH_2_- and -CH_3_ groups were attributed to the absorption peaks at 2930 cm^−1^ and 2871 cm^−1^. The characteristic peaks of the amide I band (C=O stretching vibration) and the N—H bending vibration of amino groups (−NH_2_) were at 1657 cm^−1^ and 1590 cm^−1^ respectively. Conversely, the FTIR spectrum of QCS showed significant changes. A marked enhancement in the absorption intensity around 3412 cm^−1^ was also observed for QCS, which was attributed to the increased hydrogen-bonding interactions resulting from the introduction of quaternary ammonium groups. The N—H bending vibration of amino groups was still observable at 1564 cm^−1^, indicating that some amino groups were not completely substituted. Above all, a new characteristic absorption peak appeared at 1500 cm^−1^ in QCS. The C—H bending vibration of the methyl groups of the quaternary ammonium group was attributed to this peak and is taken as a diagnostic indicator of the successful grafting of quaternary ammonium groups. Based on the combined spectral features, including the emergence of a new characteristic peak at 1500 cm^−1^, the significantly enhanced absorption at 3412 cm^−1^, and the coexistence of the residual amino peak at 1564 cm^−1^, it was strongly suggested that the quaternary ammonium groups were successfully grafted onto CS molecular chains *via* reaction with amino groups. This result demonstrated the formation of the expected partially substituted structure, thereby confirming the synthesis of QCS.

### 3.2 Formula optimization

EE and LC were applied as assessment parameters in this present study and the optimum concentrations of QCS, TPP, and CMCNa were identified using Design Expert 8.0 software. In this model, a standardized estimation of the prediction uncertainty across the entire design space required fifteen experiment runs with five repetition center points. The trial design and results generated in the Design-Expert program were presented in Table S1. Based on the analysis, the results of fifteen groups were presented in Tables S2 and S3, as well as the quadratic poly nomial regression equation between the LC and two factors:

$$\mathrm{LC}=23.36+1.14\times A+1.36\times B+0.0490\times C+0.9067\times \mathrm{AB}-0.3049\times \mathrm{AC}-0.4963\times \mathrm{BC}-1.74\times A^{2}–3.10\times B^{2}–0.5327\times C^{2}$$

The quadratic polynomial regression equation of the EE *versus* the two factors was:

$$\mathrm{EE}=+89.22+6.37\times A+8.45\times B+0.8328\times C-2.11\times \mathrm{AB}+0.9006\times \mathrm{AC}-1.22\times \mathrm{BC}-5.89\times A^{2}–10.86\times B^{2}–3.84\times C^{2}$$

**Fig. 2:**
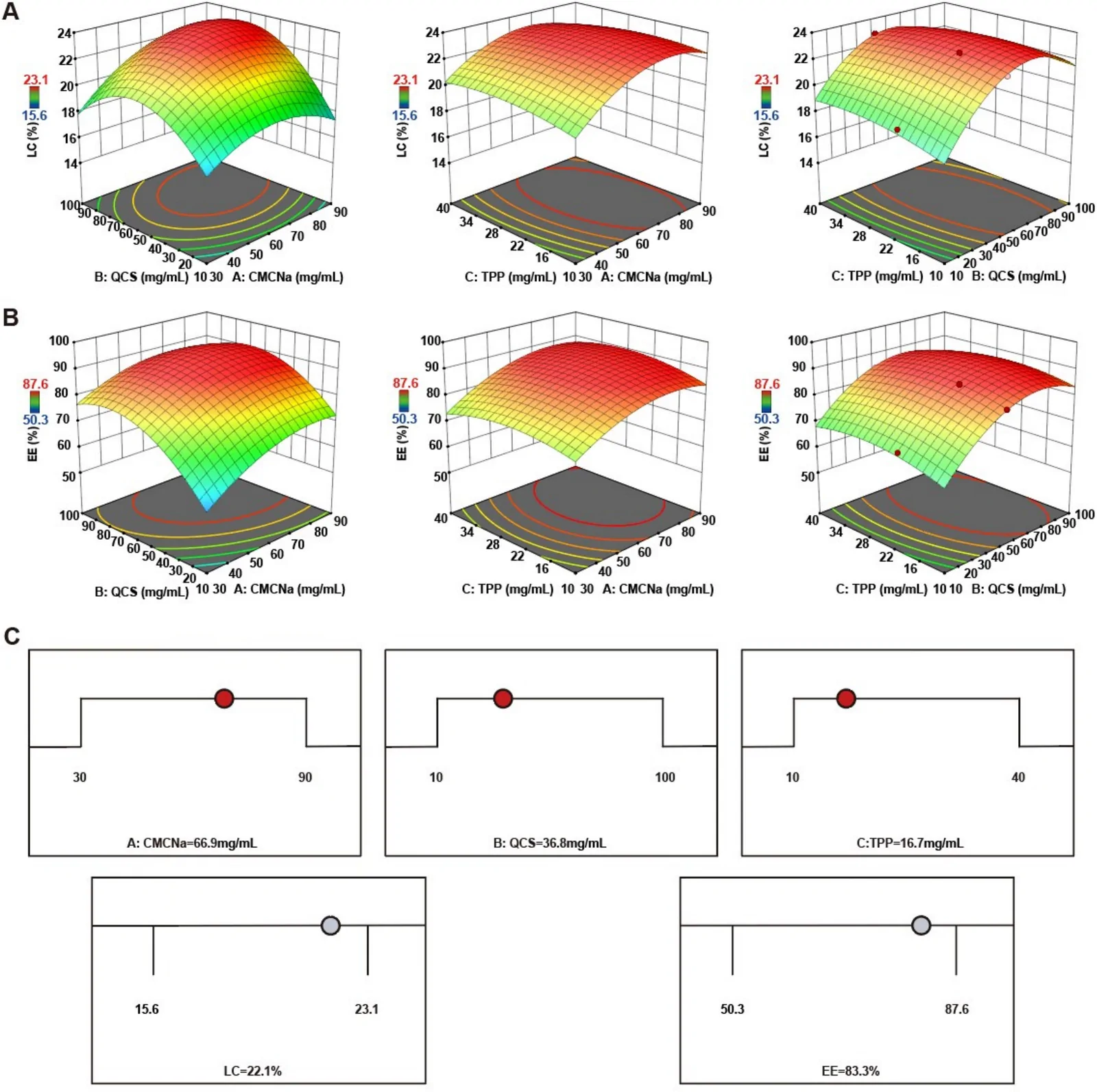
Three-dimensional layout of response surface images of the various concentrations of QCS, TPP and CMCNa to LC (A) and EE (B); C: The optimal formula calculated with the Design-Expert software.

The established quadratic polynomial model exhibited highly significant statistical parameters (LC: *P* < 0.0001, R^2^ = 0.9934; EE: *P* < 0.0001; R^2^ = 0.9918), and the predicted values were in excellent agreement with the experimental data (|Predicted R^2 _^ Adjusted R^2^| < 0.2), indicating that the model possessed good fitting accuracy and predictive reliability. Analysis of variance revealed that the concentration of QCS exerted the most significant influence on both LC and EE, which could be attributed to the strong electrostatic interaction with CMCNa. Meanwhile, a significant positive effect of CMCNa concentration was also observed, confirming that the electrostatic interaction between the hydrogel materials served as the core mechanism driving the self-assembly of the nanogels and the subsequent drug encapsulation. Notably, all quadratic terms in the model were found to be significant (*P* < 0.05), suggesting that the effects of the investigated factors on the responses were not simply linear. Instead, a threshold effect was demonstrated, whereby excessively high concentrations of a given factor might lead to a decrease in LC and EE due to excessive molecular chain entanglement or charge shielding effects. Interestingly, a significant negative interaction was identified between QCS and TPP. This phenomenon elucidated the necessity for precise regulation of the crosslinker dosage. Specifically, an excess of TPP was suggested to compete with CMCNa for binding with QCS, resulting in the formation of locally excessive ionic crosslinking points. Consequently, the hydrogel nano-network structure was transformed from a loose and porous “loading-optimal” conformation to a densely contracted “compact” conformation, which was unfavorable for the efficient encapsulation of drug molecules. In contrast, a significant positive interaction was observed between CMCNa and QCS, indicating that these two components exerted a synergistic effect at an appropriate ratio, facilitating the construction of a composite nano-gel network with a well-organized structure and enhanced loading capacity. Based on the above information, three dimensional reaction surface pictures were drawn (Fig. 2A-B). The optimal formulation, identified through response surface methodology, was determined as follows: CMCNa concentration of 66.9 mg/mL, QCS concentration of 36.8 mg/mL, and TPP concentration of 16.7 mg/mL. Under these optimized conditions, the model predicted a drug loading capacity of 22.1% and an encapsulation efficiency of 83.3%, with a desirability value of 1.000 (Fig. 2C). This optimized formulation achieved a high EE while significantly reducing the required amounts of QCS and TPP, thereby demonstrating both cost-effectiveness and the principle that “moderate crosslinking and synergistic assembly” are crucial for constructing high-capacity nanogel delivery systems. Based on the results of the box-behnken response surface optimization, and following a comprehensive consideration of model prediction accuracy alongside the practical operability of large-scale preparation, the optimal formulation for subsequent studies was definitively established as follows: 67 mg/mL CMCNa, 37 mg/mL QCS and 17 mg/mL TPP. This formulation was selected because it closely approximates the theoretically optimal ratio predicted by the model, while ensuring the structural integrity, particle size uniformity, and preparation reproducibility of the nanogel. Under these practical concentration conditions, the EE of 82.9 ± 1.4% and LC of 21.8 ± 0.3% were achieved for the triple-layered core-shell nanogel. These values were found to be in excellent agreement with the predictions of the response surface model, thereby further validating the predictive precision of the established quadratic polynomial regression model and the reliability of the optimization results.

### 3.3 Characterization

**Fig. 3:**
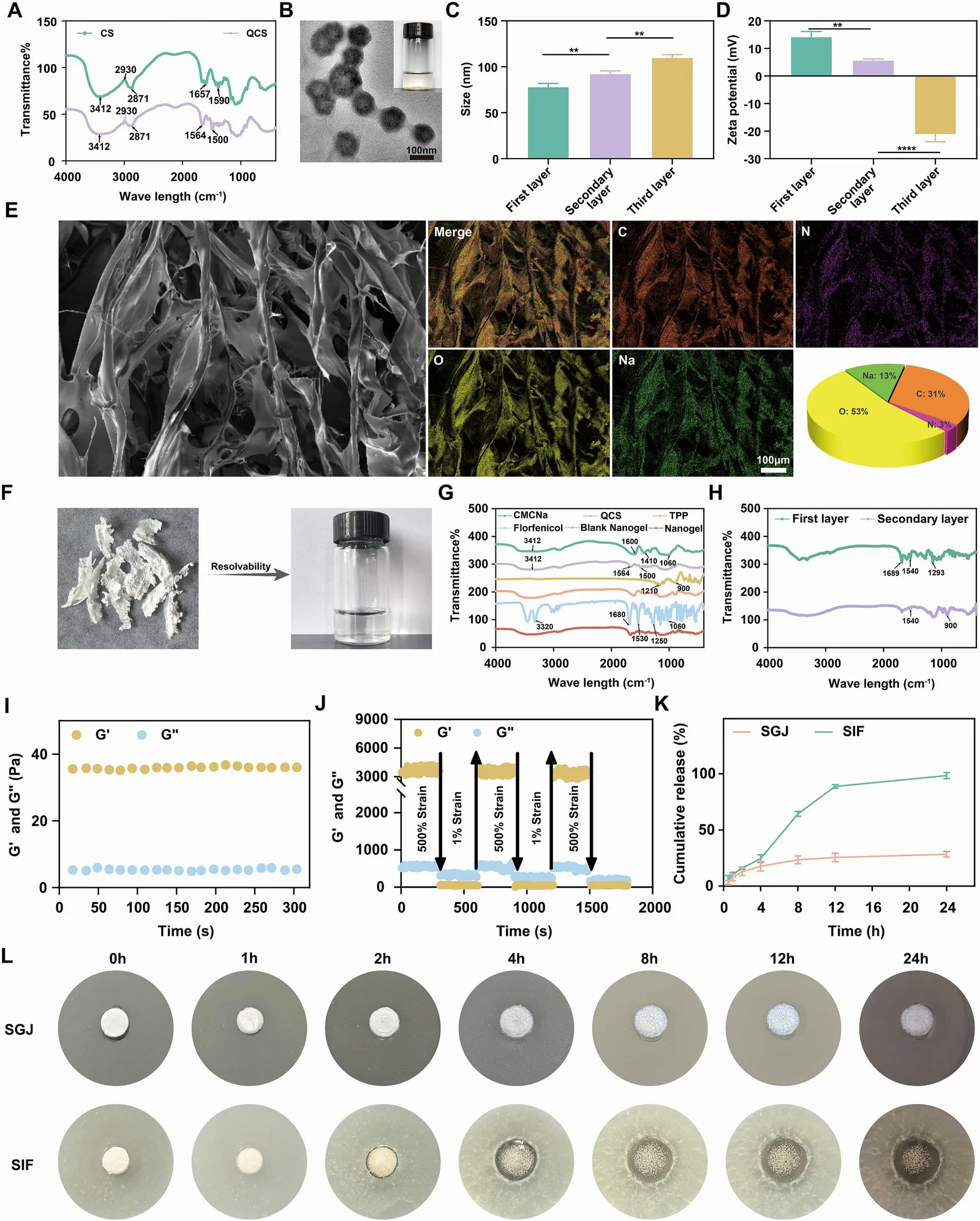
Physical and chemical characterization of the triple-layered core-shell nanogel. FTIR of QCS (A); B: Appearance and TEM; C: size; D: zeta potential; E: SEM and EDS; F: Resolvability; G-H: FTIR; Time sweep mode (I); G' and G" of nanogel in continuous step strain sweep 1–500% strain (J); K: *In vitro* release (K) and degradation behavior (L) in SGJ and SIF.

The prepared triple-layered core-shell nanogel appeared as a clear and transparent solution in PA buffer, with no flocculence or precipitation observed upon standing, indicating excellent colloidal stability and homogeneous dispersion (Fig. 3B). Observation by TEM revealed that the triple-layered core-shell nanogel were spherical in shape with uniform particle size (Fig. 3B), and the average diameter was measured to be approximately 100 nm. As shown in Fig. 3C and Fig. 3D, the first layer nanoparticles exhibited a particle size of 78.1 ± 3.0 nm and a zeta potential of 14.2 ± 1.6 mV, indicating the successful formation of a positively charged drug-polymer composite core. Following TPP cross-linking, the secondary layer nanoparticles exhibited an increased particle size of 92.3 ± 2.6 nm and a decreased zeta potential of 5.7 ± 0.4 mV. After CMCNa coating, the particle size of the tertiary nanogel further increased to 110.0 ± 2.7 nm, and the zeta potential was reversed to −21.2 ± 2.6 mV, indicating that the anionic CMCNa outer shell was successfully assembled onto the nanoparticle surface, ultimately forming a complete negatively charged triple-layered core-shell nanogel. This negative charge could effectively prevent nanoparticle aggregation through electrostatic repulsion, and avoid the non-specific cytotoxicity that might be induced by an excessively high positive charge, reflecting a favorable biocompatibility design. Following freeze-drying, the nanogel appeared as a white, fluffy solid, which could be rapidly dissolved upon the addition of water, reverting to a clear and transparent solution (Fig. 3F). This demonstrated that nanogel has excellent redispersion capability, offering convenience for long-term storage and clinical application.

SEM further revealed the formation of a typical three-dimensional porous network structure within the nanogel due to the electrostatic interaction between CMCNa and QCS, as well as the TPP-mediated ionic cross-linking. EDS revealed that C (31%), N (3%), O (53%), and Na (13%) were evenly spread across the nanogel network (Fig. 3E). The characteristic distribution of N confirmed the successful incorporation of QCS, and the elemental uniformity provided evidence, at the chemical composition level, of molecular-level homogeneous mixing and cooperative assembly among the three components. These results demonstrate that triple-layered core-shell nanogels with uniform morphology, excellent stability, an ordered structure, and favorable redispersion properties were successfully constructed in this study, laying the foundation for their application as an oral delivery system for targeted drug administration and enhanced antibacterial efficacy. FTIR spectroscopy was used to further confirm the successful fabrication of the triple-layered core-shell nanogel and the effective encapsulation of florfenicol (Fig. 3G and Fig. 3H). In the FTIR spectrum of the blank nanogels, the asymmetric stretching vibration band of the carboxylate group (COO^−^) of CMCNa, originally located at 1600 cm^−1^, was shifted to a higher wavenumber (1620 cm^−1^). At the same time, an apparent reduction in the strength of the amide II band of QCS at 1590 cm^−1^ was seen. These transformations pointed to the existence of a powerful electrostatic interaction between CMCNa and QCS, which resulted in the creation of a polyelectrolyte complex. In the meantime, the typical P=O absorption and P—O absorption bands of TPP, which were found at 1210 cm^−1^ and 1090 cm^−1^ respectively, could still be observed albeit with less intensity and a wider peak shape. This confirmed that TPP was bound to the quaternary ammonium groups of QCS *via* ionic cross-linking, collaboratively contributing to the construction of a stable triple-layered core-shell network. In the FTIR spectrum of the first layer nanoparticles, the amide II band absorption peak of QCS at 1564 cm^−1^ was red-shifted to 1540 cm^−1^ with slightly weakened intensity. Meanwhile, the amide I band absorption peak of florfenicol at 1680 cm^−1^ was red-shifted to 1689 cm^−1^, and the C—F stretching vibration peak at 1250 cm^−1^ was shifted to 1293 cm^−1^. These spectral changes indicated the formation of non-covalent interactions, such as hydrogen bonding, between florfenicol and QCS. Notably, no new characteristic absorption peaks were observed in the spectrum, confirming that no chemical bond was formed between the two components, and that florfenicol was primarily encapsulated within the first layer nanoparticles *via* physical entrapment. The FTIR spectrum of the secondary layer nanoparticles revealed the emergence of characteristic P=O (1210 cm^−1^) and P—O (900 cm^−1^) absorption bands, accompanied by a marked reduction in the intensity of the amide II band of QCS at 1540 cm^−1^, confirming the formation of an ionic cross-linking intermediate layer between TPP and the quaternary ammonium groups of QCS. In the spectrum of the triple-layered core-shell nanogel, the characteristic absorption bands of florfenicol, originally located at 1680 cm^−1^ (amide I band) and 1520 cm^−1^ (amide II band), exhibited a band shift to approximately 1690 cm^−1^ with diminished intensity. The C—F stretching vibration band at 1250 cm^−1^ was also shifted. These observations demonstrated that florfenicol was successfully encapsulated within the nanogel core and that non-covalent interactions, such as hydrogen bonding, were formed between the drug and the carrier matrix. Notably, no new characteristic absorption bands were detected in the spectrum of the drug-loaded nanogels, which ruled out the possibility of chemical bond formation between the drug and the carriers. It was therefore confirmed that florfenicol was primarily embedded within the nanogels through physical encapsulation. Collectively, these FTIR results confirmed that the triple-layered core-shell nanogel was successfully constructed *via* the synergistic effects of electrostatic self-assembly and ionic cross-linking.

The mechanical properties and structural stability of the triple-layered core-shell nanogel were further evaluated by rheological analysis. As revealed by the time sweep curve, G' remained stable at approximately 35 Pa throughout the entire testing period (0–300 s), while G" was maintained at around 5 Pa. No significant fluctuations or attenuation were observed in either modulus, demonstrating the excellent structural stability of the nanogel system, as no network disruption or rearrangement occurred under continuous shear. More importantly, G' was consistently and significantly higher than G", a characteristic behavior of typical hydrogel materials, thereby confirming the formation of an elastic-dominated three-dimensional network structure (Fig. 3I). Further investigation *via* strain/frequency sweep analysis revealed that under broader testing conditions, G' was maintained above 3000 Pa, while G" was approximately 200 Pa, resulting in a G'/G" ratio as high as 15 (Fig. 3J). This indicated that the nanogel possessed high-strength and high-elasticity mechanical characteristics. This superior mechanical performance can be attributed to the dense three-dimensional network constructed by the electrostatic interaction between CMCNa and QCS, as well as the TPP-mediated ionic cross-linking. Collectively, these rheological properties provide a reliable mechanical foundation for the triple-layered core-shell nanogel to resist gastrointestinal peristaltic shear forces, maintain structural integrity, and achieve targeted release during oral delivery.

In this study, the *in vitro* release behavior of the triple-layered core-shell nanogel was investigated in SGJ (pH 1.2) and SIF (pH 7.4), and the cumulative drug release profiles were presented in Fig. 3K. Significant differences were observed in the release behavior of florfenicol-loaded nanogels between the two media, fully demonstrating the pH-responsive release characteristics of this delivery system upon exposure to the gastrointestinal environment. In SGJ, the release kinetics of the triple-layered core-shell nanogel was relatively slow, with a cumulative release of only 28.4 ± 2.4% at 24 h. The sustained release profile was governed by the structural stability of the nanogels under acidic conditions. Under low pH conditions, protonation of the carboxylate groups of CMCNa occurred, leading to weakened electrostatic interactions with the quaternary ammonium groups of QCS. However, hydrogen bonding interactions were enhanced, and the TPP-crosslinked network remained relatively stable in the acidic environment. Collectively, a more compact three-dimensional network structure was formed, which effectively delayed the diffusion and release of drug molecules. This result indicated that the triple-layered core-shell nanogel significantly inhibited the early release of the drug in the gastric environment, thereby reducing drug degradation and loss in the stomach, providing favorable conditions for targeted release upon entry into the intestine. In SIF, typical pH-responsive release behavior was exhibited by the triple-layered core-shell nanogel. The cumulative release was only 24.5 ± 3.4% during the initial phase (0–4 h), with no obvious burst release observed, indicating that the nanogel maintained good structural integrity at the early stage in SIF, thereby avoiding extensive drug leakage. Subsequently, a rapid release phase was entered (4–12 h), during which the cumulative release rapidly increased from 24.5 ± 3.4% to 88.7 ± 1.6%, with a markedly accelerated release rate. This rapid release behavior was attributed to the structural alteration of the nanogel triggered by SIF. Under pH 6.8 conditions, complete dissociation of the carboxylate groups of CMCNa occurred, resulting in enhanced electrostatic interactions with the quaternary ammonium groups of QCS. However, network swelling and increased porosity may also have been induced by changes in ionic strength, thereby accelerating drug diffusion and release. During the later stage (12–24 h), the release rate gradually decreased, and the cumulative release further accelerated from 88.7 ± 1.6% to 98.4 ± 2.7%, indicating that the drug was almost completely released, with minimal residual amount. Notably, the differences in release behavior observed in the two media clearly demonstrated the pH-responsive release characteristics of the triple-layered core-shell nanogel. In the acidic gastric environment (pH 1.2), a compact structure was maintained by the nanogel, resulting in slow drug release (only 28.4 ± 2.4% at 24 h), which effectively protected the drug from degradation by gastric acid and pepsin. Upon entry into the weak alkaline intestinal environment (pH 6.8), structural responsiveness was exhibited by the nanogels, with a significantly accelerated release rate, and a large amount of drug was released within 4–12 h (cumulative release rate of 88.7 ± 1.6%), facilitating rapid drug absorption during the intestinal absorption window. In addition, the results showed that no significant degradation were observed in the nanogel within 24 h in SGJ, indicating favorable structural stability, whereas structural disintegration was initiated after 4 h in SIF, confirming that the triple-layered core-shell nanogel could be gradually degraded in the intestinal environment (Fig. 3L). This degradation behavior was found to be highly consistent with the aforementioned “sustained release in the stomach and accelerated release in the intestine” release profile, jointly demonstrating that the nanogel could maintain its structural integrity in the stomach to protect the encapsulated drug, while being gradually degraded in the intestine to facilitate drug release and metabolic clearance. Collectively, these findings provide dual structural and functional assurance for gastric protection, targeted intestinal release, and *in vivo* safety during oral administration.

### 3.4 Stability assessment

**Fig. 4:**
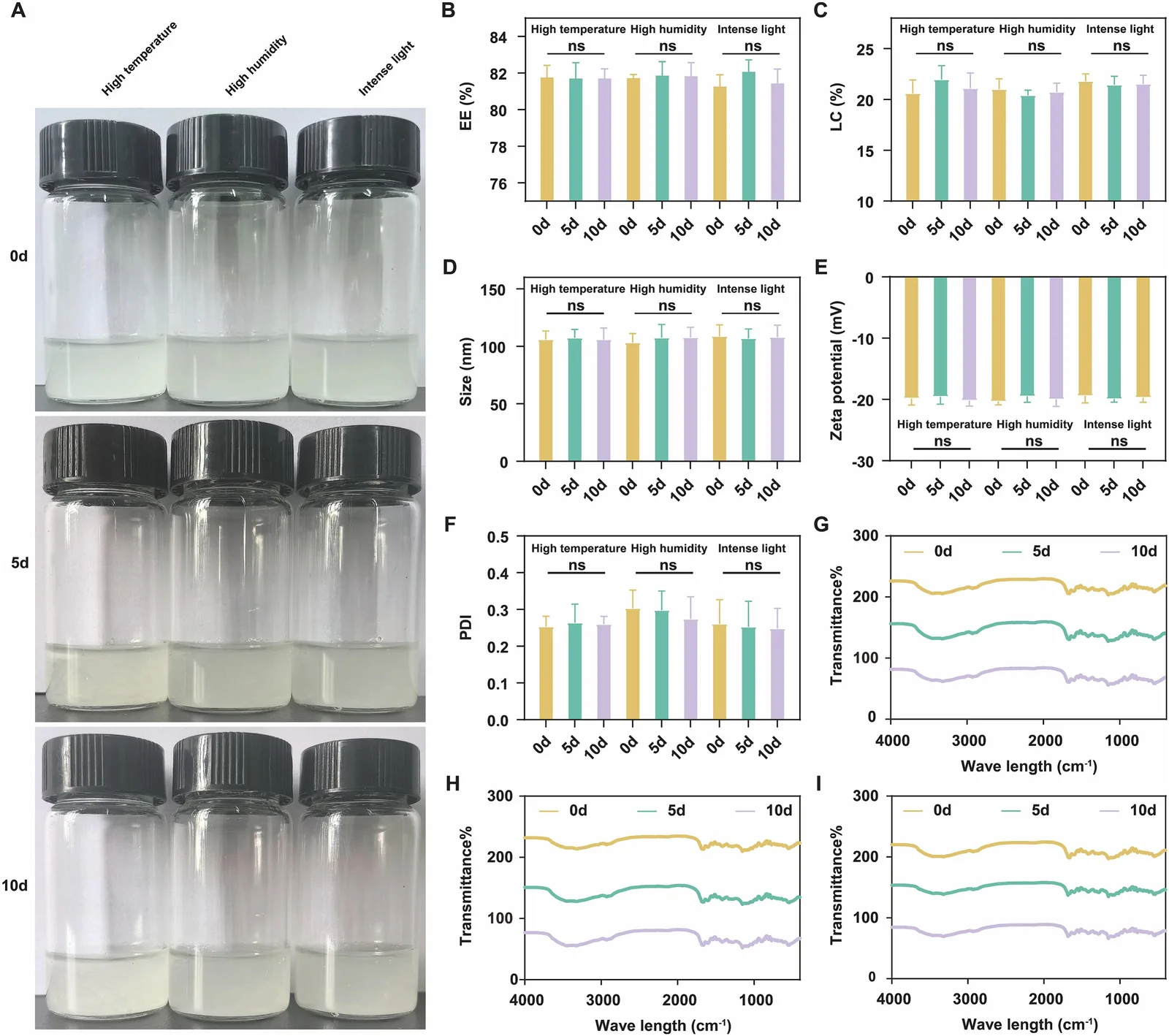
The influence factors test (high temperature, high humidity, and intense light) of triple-layered core-shell nanogel. Appearance (A); EE (B); LC (C); size (D); ZP (E); PDI (F); FTIR spectrophotometer (G: high temperature, (H) high humidity, (I) intense light).

To assess the stability of the triple-layered core-shell nanogel, a series of influencing-factor studies were conducted under defined stress conditions, including high temperature (40 °C), high humidity (90% ± 5% RH), and intense light (4500 ± 500 lx). The evaluation was performed based on multiple parameters, including macroscopic appearance, EE, LC, particle size, ZP, PDI, and FTIR spectroscopy. Throughout the 10-day observation period, no significant alterations were detected in any of the evaluated indicators on either day 5 or day 10. The triple-layered core-shell nanogel consistently exhibited a clear and transparent solution in PA buffer (Fig. 4A). Quantitative analyses revealed that EE remained stable at approximately 82% (Fig. 4B), LC at around 21% (Fig. 4C), particle size at roughly 110 nm (Fig. 4D), ZP at approximately −21 mV (Fig. 4E), and PDI at about 0.25 (Fig. 4F). Furthermore, FTIR spectral profiles showed no significant differences across the tested time points (Fig. 4G-I). Collectively, these findings demonstrate that the prepared the triple-layered core-shell nanogel possessed satisfactory stability under the evaluated stress conditions, supporting their potential for further pharmaceutical development and application.

### 3.5 Enhanced antibacterial activity

**Fig. 5:**
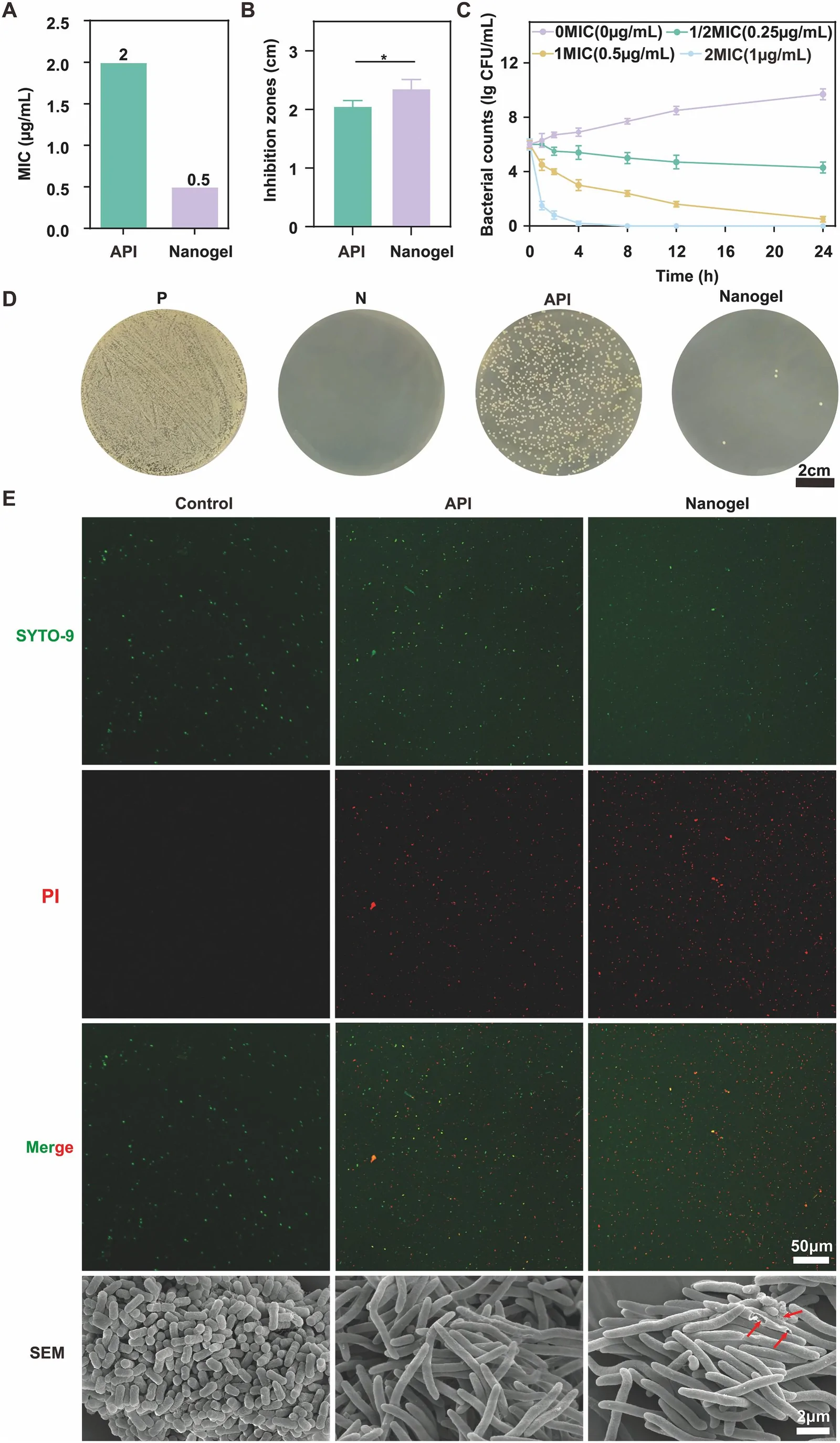
The antibacterial effect of the triple-layered core-shell nanogel against *E. coli*. MICs (A); Inhibition zones (B); *In vitro* killing curves (C); Colony counting (D); Live/dead bacterial staining and SEM image (E).

The MIC of florfenicol API and the triple-layered core-shell nanogel against the *E. coli* isolate and *S. aureus* isolate were characterized using the broth macrodilution method. The MIC of florfenicol API against the *E. coli* isolate was determined to be 2 μg/mL, whereas that of the triple-layered core-shell nanogel was significantly reduced to 0.5 μg/mL, indicating a fourfold enhancement in antibacterial activity conferred by the nano-delivery system (Fig. 5A). Similarly, against the *S. aureus* isolate, the MIC of the nanogel decreased from 4 μg/mLL (API) to 1 μg/mL, further confirming the broad-spectrum antibacterial potency of the formulation (Fig. S1A). Consistent with the MIC results, the inhibition zone diameter of the triple-layered core-shell nanogel against *E. coli* isolate (2.36 ± 0.13 cm) was significantly larger than that of the free florfenicol API (2.06 ± 0.08 cm, *P* < 0.05) (Fig. 5B). Similarly, against *S. aureus* isolate, the nanogel also exhibited a significantly larger inhibition zone (2.22 ± 0.11 cm) compared with the API group (1.93 ± 0.06 cm, *P* < 0.05) (Fig. S1B). These results collectively confirmed that the encapsulation of florfenicol into the triple-layered core-shell nanogel significantly enhanced its inhibitory activity against both Gram-negative and Gram-positive bacteria. The *in vitro* time-kill curves of different concentrations of triple-layered core-shell nanogel (0×, 1/2×, 1×, and 2 × MIC) against the *E. coli* isolate and *S. aureus* isolate were further evaluated. When treated with triple-layered core-shell nanogel at 2 × MIC (1 μg/mL), the bacterial count dropped below the detection limit after 4 h of treatment and was maintained in a completely inhibited state for 24 h. Partial inhibitory effects were observed at concentrations of 1/2 × MIC and 1 × MIC (Fig. 5C and Fig. S1C). Therefore, the excellent bactericidal effect of the triple-layered core-shell nanogel against the *E. coli* isolate and *S. aureus* isolate became more pronounced with increasing concentration, demonstrating the triple-layered core-shell nanogel exhibited concentration-dependent characteristics. To directly compare the antibacterial efficacy of florfenicol API and the triple-layered core-shell nanogel, the survival status of bacteria treated with an equal concentration (1 × MIC) of drugs was quantitatively assessed using the plate counting method. Following the treatment with the triple-layered core-shell nanogel, very few single colonies were formed upon plating. In contrast, a substantial number of colony formations were still observed on the plates treated with an equal concentration of florfenicol API. Therefore, the triple-layered core-shell nanogel exhibited stronger antibacterial activity than florfenicol API, which might be attributed to the nano-delivery system improving the drug's water dispersibility, bacterial membrane permeability, and local retention effect (Fig. 5D and Fig. S1D). Finally, SEM was employed to further observe the morphological alterations of both *E. coli* and *S. aureus* isolates following different treatments. In the negative control group, *E. coli* exhibited a typical short-rod morphology with a smooth, plump surface and intact cell membranes, while *S. aureus* displayed the characteristic spherical shape with a uniformly smooth and intact surface. Following treatment with florfenicol API, *E. coli* showed a tendency to elongate and thin with only minor surface damage, whereas *S. aureus* appeared shrunken and distorted, with some loss of structural integrity. In marked contrast, the triple-layered core-shell nanogel treatment induced far more pronounced morphological changes in both bacterial strains. Most *E. coli* exhibited abnormal elongation, pronounced depressions, folds, and even large-area perforations on the cell surface, with clear signs of intracellular content leakage in some cells. Similarly, *S. aureus* displayed extensive rupture, lysis, and obvious leakage of cytoplasmic contents, indicating severe membrane disruption. These morphological differences strongly suggest that the triple-layered core-shell nanogel is more effective in compromising the integrity of the bacterial cell membrane or cell wall, thereby accelerating bacterial death (Fig. 5E and Fig. S1E). This enhanced bactericidal effect may be attributed to the cationic nature of the QCS component, which imparts excellent bacterial membrane affinity to the nanogel and facilitates the efficient delivery of florfenicol to its intracellular targets.

### 3.6 Obvious oral delivery and biodistribution

**Fig. 6:**
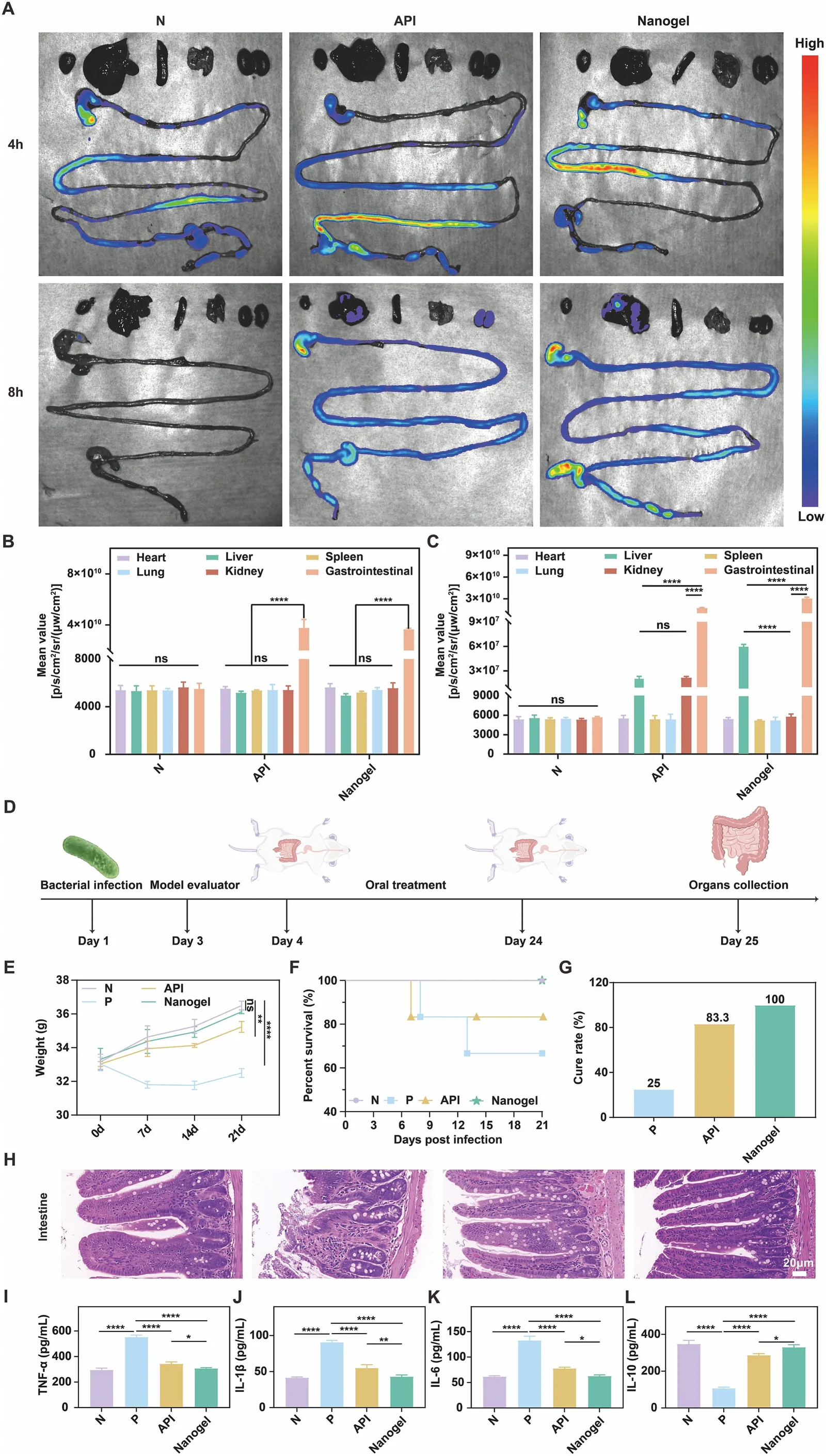
*In vivo* biodistribution and therapeutic efficacy of the triple-layered core-shell nanogel. A: Fluorescence images of mice after oral administration of PBS, API and nanogel; Fluorescence intensity of each tissue (heart, liver, spleen, lung, kidney and gastrointestinal tract) at 4 (B) and 8 (C) h; D: Treatment schedules; E: Weight change; F: survival rate; G: Cure rate; H: H&E; Measurement of TNF-α (I), IL-1β (J), IL-6 (K) and IL-10 (L) in the intestine.

To evaluate the *in vivo* oral delivery and biodistribution of the triple-layered core-shell nanogel, the distribution of the drugs (API and nanogel) in the major organs and gastrointestinal tract of mice following oral administration was investigated using DiR fluorescence labeling and *in vivo* imaging system, with PBS administered as a negative control (Fig. 6A). In the negative control group, no detectable fluorescence signals were detected in vital organs (heart, liver, spleen, lungs, kidneys, or gastrointestinal tract) at any time point (4 and 8 h), with extremely low fluorescence intensity observed. The signals of strong fluorescence were found in the gastrointestinal tract of the free API group and the nanogel group at 4 h post-administration and the fluorescence intensities were significantly stronger than that of the negative control group, which indicated that both formulations readily penetrated the gastrointestinal tract following oral administration and produced effective distribution. Notably, no obvious fluorescence was observed in the major organs (heart, liver, spleen, lung and kidney) at 4 h, with extremely low fluorescence intensity observed, suggesting that the drugs (API and nanogel) were primarily confined to the gastrointestinal tract during the initial phase, with no significant systemic absorption or marked translocation to distant organs (Fig. 6B). At 8 h post-administration, a decrease in fluorescence intensity was observed in the gastrointestinal tract of the free API group compared to that at 4 h, indicating that partial drug clearance or translocation from the gastrointestinal tract had occurred. Concurrently, significantly increased fluorescence signals were observed in the liver and kidney, suggesting that after absorption from the gastrointestinal tract, the API was primarily metabolized and excreted *via* the liver and kidney. However, no obvious fluorescence intensities were observed in the heart, spleen or lung, indicating that no specific accumulation of the API occurred in these tissues. In contrast, for the triple-layered core-shell nanogel group at 8 h, no significant attenuation of fluorescence intensity in the gastrointestinal tract was observed compared to that at 4 h, with a consistently high level maintained, indicating superior retention capacity of the nanogel in the gastrointestinal tract (Fig. 6C). This retention was likely attributed to the mucoadhesive properties of QCS and the anti-clearance capability conferred by the triple-layered core-shell nanogel. Similar to the API group, an increased fluorescence intensity was also detected in the liver of the nanogel group. However, no obvious enhancement in fluorescence intensity was detected in the kidney, suggesting that after oral absorption, the nanogel were predominantly distributed in the liver with limited distribution in the kidney. This distribution pattern may be related to their relatively large particle size and surface charge characteristics. No obvious fluorescence signal intensity was detected in the heart, spleen or lung. In summary, compared with the API, the triple-layered core-shell nanogel exhibited superior gastrointestinal retention capacity after oral administration, with no significant attenuation of fluorescence intensity in the gastrointestinal tract observed at 8 h. Additionally, a distinct distribution pattern was observed in the major metabolic organs (liver and kidney), particularly with reduced distribution in the kidneys, suggesting that prolonged systemic circulation time may be achieved through decreased renal excretion. These findings support the potential use of the triple-layered core-shell nanogel as an oral delivery system for localized intestinal targeting and sustained release.

### 3.7 Outstanding therapeutic efficacy

The *in vivo* therapeutic efficacy of the triple-layered core-shell nanogel was systematically evaluated using a mouse model of *E. coli* isolate infection (Fig. 6D). The triple-layered core-shell nanogel treatment group exhibited significantly superior outcomes in terms of body weight change, survival rate and cure rate compared to florfenicol API group. Regarding body weight recovery, a marked acceleration was observed in the triple-layered core-shell nanogel-treated group from day 14 post-treatment onwards. Following 21 days of treatment, the body weight of mice in this group (36.1 ± 0.09 g) had reached a value not markedly different to that of the negative control group (36.5 ± 0.21 g, *P* > 0.05). Conversely, the body weight of mice in the florfenicol API-treated group (35.2 ± 0.26 g) was still substantially lower than that of the negative control group (*P* < 0.01), which suggests that the triple-layered core-shell nanogel delivery system was more effective in the reversal of infection-induced systemic metabolic disorders (Fig. 6E). Dynamic monitoring of survival rates further confirmed the therapeutic advantage of the triple-layered core-shell nanogel. A 100% survival rate was maintained in the nanogel treatment group from day 6 post-infection until the experimental endpoint, whereas the survival rates in the florfenicol API-treated group and the positive infection group were 83.3% and 66.7%, respectively (Fig. 6F). Most importantly, a cure rate of 100% was achieved in the triple-layered core-shell nanogel-treated group, which was considerably higher than the 83.3% observed in the florfenicol API-treated group (Fig. 6G). This finding strongly demonstrated that the triple-layered core-shell nanogel delivery system significantly enhanced the *in vivo* therapeutic efficacy of florfenicol.

Histopathological examination revealed that the intestinal tissue structure in the triple-layered core-shell nanogel treatment group was largely intact, with neatly arranged villi and only sparse, scattered inflammatory cell infiltration compared to both the negative and positive control groups. Although some improvement was noted in the API-treated group relative to the positive infection group, focal epithelial damage and mild edema were still observed (Fig. 6H). These findings provide histological evidence further validating the protective effect of the triple-layered core-shell nanogel against infectious intestinal injury. The observed therapeutic advantages can be attributed to the multiple synergistic mechanisms conferred by the triple-layered core-shell nanogels. Firstly, QCS, acting as a cationic antibacterial agent, binds to the surface of *E. coli* through electrostatic adsorption, thereby disrupting the bacterial outer membrane integrity and facilitating increased membrane permeability. This “membrane-disrupting" effect not only exerted a direct bactericidal action but also created channels that enable the entry of florfenicol into the bacterial cell. This enabled florfenicol to efficiently reach its ribosomal target and exert its protein synthesis inhibitory function. Consequently, a dual synergistic antibacterial mechanism, characterized by “membrane disruption coupled with protein synthesis inhibition,” was established between QCS and florfenicol, leading to a significantly enhanced overall bactericidal effect against *E. coli*. Secondly, the triple-layered core-shell nanogels endows the nanogels with superior *in vivo* targeted delivery performance. On the one hand, the mucoadhesive properties mediated by QCS enable the precise accumulation of the triple-layered core-shell nanogels at the intestinal infection site, achieving targeted delivery of florfenicol. This resulted in an increased local drug concentration while reducing systemic side effects. On the other hand, their nanoscale particle size (110.0 ± 2.7 nm) conferred excellent tissue penetration capability, allowing deep infiltration into the intestinal villus spaces and deep within the infection foci, thereby delivering the drug directly to bacterial colonization sites. This dual advantage of “mucoadhesion coupled with deep penetration” was unattainable with florfenicol API. Furthermore, in terms of release characteristics, the outermost TPP-crosslinked network facilitated a sustained release of florfenicol at the infection site. An initial burst release was effectively avoided, and a consistently effective drug concentration was maintained, thereby ensuring a prolonged antibacterial effect. In summary, the *in vivo* therapeutic potential of florfenicol against *E. coli*-infected mice was significantly enhanced by the triple-layered core-shell nanogels through a triple mechanism involving: synergistically enhanced antibacterial action achieved by component cooperation, targeted delivery and penetration enabled by the delivery system, and controlled release realized by the structural design. This study showed a novel oral nano-antimicrobial formulation for veterinary use in the treatment of *E. coli* induced intestinal infections, demonstrating promising potential for clinical application.

The dynamic changes in inflammatory cytokines serve as important indicators for evaluating the therapeutic efficacy and immunomodulatory effects of the triple-layered core-shell nanogel. The concentrations of pro-inflammatory (TNF-α, IL-1β and IL-6) and anti-inflammatory (IL-10) cytokines in the intestine were measured in this research, in order to compare systematically the regulatory effects of florfenicol API and the triple-layered core-shell nanogel on the *E. coli* infection-induced inflammatory response (Fig. 6I-L). The intestinal levels of TNF-α, IL-1β and IL-6 were elevated in the positive infection group as compared to the negative control group by about 1.9 and 2.2 and 2.2-fold, respectively, and the IL-10 level was dramatically reduced by about 69.1%, which showed that the infection was effective in inducing a pro-inflammatory-dominant “cytokine storm” state. Following treatment with florfenicol API, the levels of the aforementioned pro-inflammatory cytokines were significantly decreased compared with those in the positive infection group, but were not restored to normal levels. Notably, significant differences were still observed for IL-1β (55.2 ± 3.6 pg/mL *vs.* 90.6 ± 2.2 pg/mL) and IL-10 (286.7 ± 7.1 pg/mL *vs.* 107.7 ± 4.2 pg/mL) when compared with the negative control group (*P* < 0.05). Although florfenicol API could indirectly alleviate inflammatory stimulation by inhibiting bacterial growth, its limited antibacterial activity and short duration of action *in vivo* might have been insufficient to completely eliminate the pathogenic bacteria, allowing residual bacteria to continuously activate the immune system, thereby preventing the complete resolution of the inflammatory response. In contrast, a markedly superior anti-inflammatory regulatory effect was exhibited by the triple-layered core-shell nanogel treatment group. In this group, the intestinal levels of TNF-α (307.2 ± 4.9 pg/mL), IL-1β (43.1 ± 1.9 pg/mL), and IL-6 (62.8 ± 1.9 pg/mL) were restored to levels not markedly different from those of the negative control group (*P* > 0.05), while the IL-10 level (330.1 ± 10.8 pg/mL) was also increased to near-normal levels. These findings indicated that the triple-layered core-shell nanogel not only more effectively inhibited the excessive secretio of pro-inflammatory cytokines (TNF-α, IL-1β and IL-6) but also promoted the recovery of the anti-inflammatory cytokine (IL-10), thereby re-establishing the pro-inflammatory/anti-inflammatory balance. This superior anti-inflammatory regulatory effect can be attributed to the multiple synergistic mechanisms conferred by the triple-layered core-shell structure. First, the triple-layered core-shell nanogel killed bacteria through their excellent antibacterial activity, thereby inhibiting the elevated release of pro-inflammatory cytokines. Second, the mucoadhesive properties mediated by QCS and the sustained release characteristics conferred by TPP cross-linking ensured targeted accumulation and prolonged action of the drug at the infection site. Moreover, the QCS element *per se* might have been actively stimulated to express the anti-inflammatory cytokine IL-10 by polarizing macrophages to the M2 phenotype, thus playing a role in restoring the pro-inflammatory/anti-inflammatory imbalance.

### 3.8 Excellent biosafety

The clinical translation of drug is determined by the therapeutic efficacy and is closely associated with the biosafety. In this study, the biosafety of the triple-layered core-shell nanogel was systematically evaluated through *in vitro* hemocompatibility assessment, *in vivo* short-term oral toxicity tests, and analysis of inflammatory cytokine responses.

**Fig. 7:**
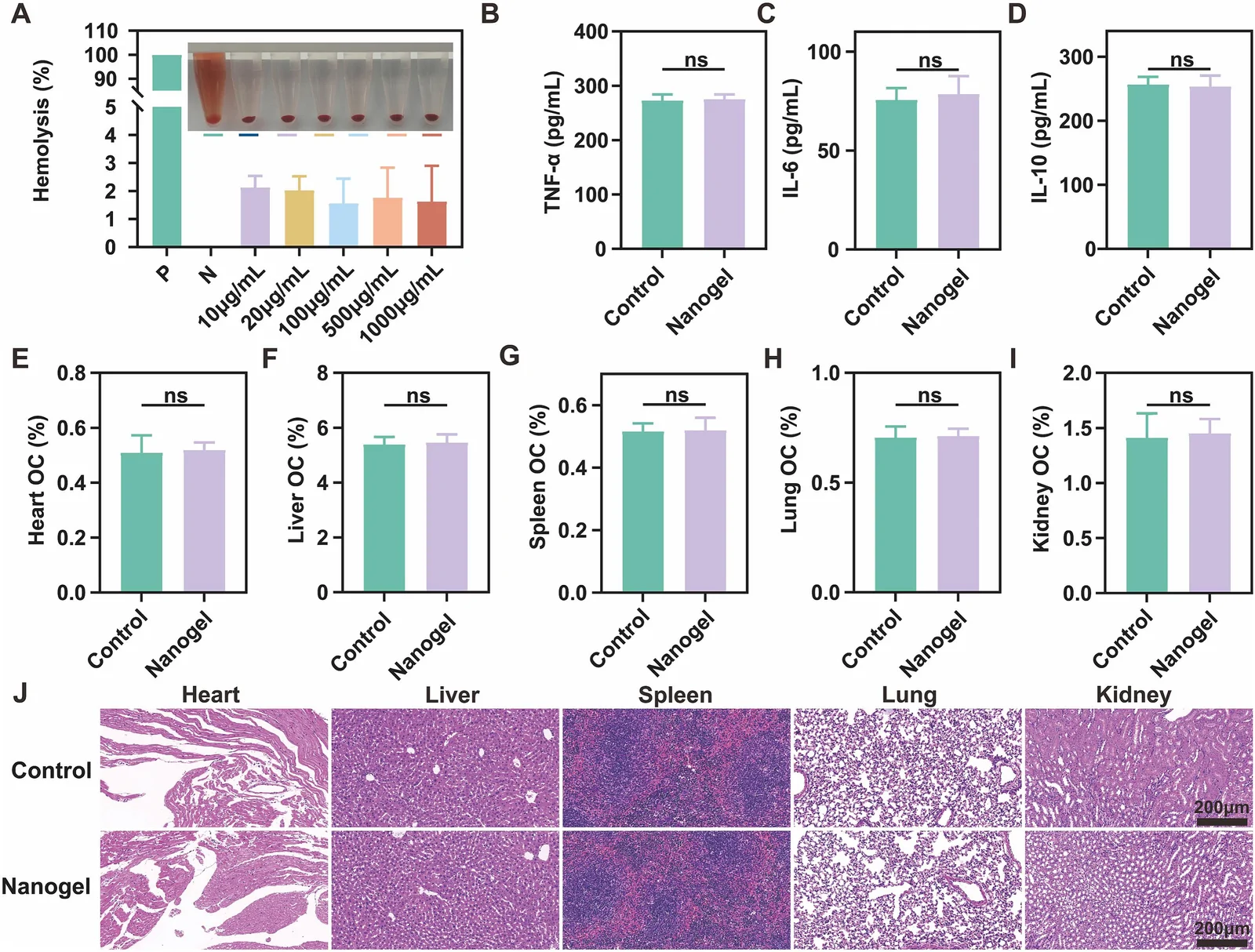
*In vitro* and *in vivo* biosafety studies. A: Hemolytic analysis; B-D: Serum levels of TNF-α, IL-6 and IL-10; *E*-I: OCs; J: histological analysis (heart, liver, spleen, lung, and kidney).

Upon intravenous or oral administration, the triple-layered core-shell nanogel may come into contact with blood cells. Therefore, hemocompatibility is considered a primary indicator in safety evaluation. In this study, the potential of the triple-layered core-shell nanogel to induce erythrocyte membrane damage was assessed using RBC hemolysis test. The findings indicated that the supernatant of the nanogel treated group was clear and transparent and there was no hemoglobin leakage as compared to the negative control group (normal saline). However, the positive control group (ultrapure water) displayed clear red hemolysis (Fig. 7A). Quantitative data revealed that the triple-layered core-shell nanogel group (0.2%) rate of hemolysis was significantly lower than the international safety standard of 5% when using biomaterials, and there was no statistically significant difference between the nanogel group and the negative control group (*P* > 0.05) (Fig. 7A). These findings demonstrated that the prepared triple-layered core-shell nanogel does not induce cell membrane disruption upon contact with blood cells, indicating favorable hemocompatibility. This observation appears to suggest the absence of concentration dependence and potentially indicates a pronounced antifouling capability. The excellent biosafety observed can be attributed to the hydrophilic shielding effect and negative charge repulsion conferred by the outer CMCNa shell, which effectively resisted non-specific adsorption of serum proteins onto the nanoparticle surface, thereby avoiding electrostatic interactions and membrane damage between the nanoparticles and erythrocyte membranes, even at high concentrations. Meanwhile, the positive charges of QCS were effectively shielded by the TPP cross-linked network and the CMCNa outer shell within the intact triple-layered core-shell structure, such that increased concentration did not lead to an elevated risk of hemolysis. To further confirm the long-term safety of the triple-layered core-shell nanogel *in vivo*, the effects of the nanogel on OCs and histomorphology in mice were systematically monitored after 14 days of continuous oral administration by gavage. The mice were weighed after the administration period and the organs (heart, liver, spleen, lung and kidney) were weighed and the OCs calculated. The findings revealed that there were no statistically significant differences in the OCs of nanogel-treated group and the control group (*P* > 0.05) (Fig. 7E-I). As OCs are considered sensitive indicators of drug-induced cumulative toxicity as well as organ edema or atrophy, these findings suggested that the triple-layered core-shell nanogel did not induce pathological damage to the organs. Subsequently, histomorphological evaluation of the organs from mice in each group was performed using H&E staining (Fig. 7J). In the negative control group, the tissue structures of all major organs were clearly defined, with normal cell morphology and no evidence of pathological changes, such as inflammatory infiltration, necrosis, or fibrosis. Notably, the histological morphology of the heart, liver, spleen, lungs and kidneys in the nanogel-treated group was closely related to the histological morphology of the control group. The hepatic cords were regularly arranged, with clear sinusoidal structures and no hepatocellular swelling or vacuolar degeneration; the glomeruli and renal tubules exhibited intact structures, with no interstitial congestion or inflammatory cell infiltration; myocardial fibers were regularly aligned, and no alveolar wall thickening was observed in the lung tissue. These findings indicated that following continuous oral administration of the triple-layered core-shell nanogel for 14 days, the structural integrity and functional stability of the organs in mice were maintained, with no significant tissue damage observed. Furthermore, the biosafety of the triple-layered core-shell nanogel was further supported by the results of blood physiological and biochemical analyses. In mice, the nanogel did not induce any significant changes in any of the indicators that were measured after the administration of the nanogel, relative to the negative control group. It is interesting to note that normal levels of white blood cells as well as the difference counts indicated that the nanogel did not cause systemic inflammatory reactions or immune rejection (Table S4 and S5). Finally, ELISA kits were used to measure the serum concentrations of TNF-α, IL-6 and IL-10 to determine the presence of a systemic immune response caused by the nanogel (Fig. 7B-D). The findings indicated that there were no statistically significant differences in the serum level of TNF-α, IL-6, and IL-10 in the nanogel-treated group of mice and that of the negative control group (*P* > 0.05). These findings confirmed that the prepared the triple-layered core-shell nanogel does not activate macrophages or induce a systemic inflammatory response, demonstrating favorable immunocompatibility.

Therefore, the triple-layered core-shell nanogel constructed in this study demonstrated excellent biosafety at both *in vitro* and *in vivo*. Firstly, this may be attributed to the inherent safety of the materials themselves. CMCNa, as a natural polysaccharide derivative, exhibits good biodegradability and low immunogenicity. Although QCS is a cationic polymer, its positive charges were effectively neutralized by the negative charges of CMCNa within the composite system, thereby avoiding excessive disruption of the cell membrane by free cationic groups. Secondly, this may be ascribed to the stability of the triple-layered core-shell nanogel. The triple-layered core-shell nanogel, formed through TPP crosslinking, effectively prevented the burst leakage of the drug before reaching the intended target site, reducing the toxic exposure of normal tissues to free drugs. Consequently, the triple-layered core-shell nanogel constructed in this study, while maintaining high antibacterial activity, significantly reduced the risk of *in vivo* toxicity, thereby establishing the robust safety basis for its further application in the field of veterinary antibacterial agents.

## 4 Conclusions

In this study, a triple-layered core-shell nanogel composed of CMCNa, QCS, and TPP was successfully constructed for the oral targeted delivery and sustained release of florfenicol. The prepared nanogel exhibited excellent colloidal stability, pH-responsive release behavior, and concentration-dependent antibacterial activity *in vitro*, exerting synergistic bactericidal effects by disrupting the integrity of the bacterial cell membrane. *In vivo* experiments further demonstrated that the nanogel possessed superior gastrointestinal retention capacity, achieving satisfactory survival rate and cure rate in infected mice while effectively regulating the pro-inflammatory/anti-inflammatory cytokine balance, thereby providing an efficient and safe nanoplatform for the oral treatment of bacterial enteritis.

## Animal welfare and ethical statement

The authors confirm that they have adhered to international standards for the protection of animals used for scientific purposes. The use of mice and all the experimental protocols concerning the handling of mice were complied with, either the ARRIVE guideline and carried out by the National Research Council's Guide for the Care and Use of Laboratory Animals (approved number: 2023008).

## CRediT authorship contribution statement

**Tianhui Wang:** Methodology. **Xingwang Ge:** Methodology. **Yidan Zhang:** Methodology. **Zhiyi Ge:** Data curation, Conceptualization. **Aizhi Han:** Methodology, Conceptualization. **Samah Attia Algharib:** Writing – review & editing, Writing – original draft. **Muhammad Usman Ghani:** Writing – review & editing, Writing – original draft. **Shuo Han:** Funding acquisition, Conceptualization. **Xiuge Gao:** Funding acquisition, Conceptualization. **Wanhe Luo:** Writing – review & editing, Writing – original draft, Funding acquisition, Conceptualization.

## Declaration of competing interest

The authors declare no conflicts of interest.

## Data Availability

The data employed to corroborate the findings of this study are available from the corresponding author upon request.

## Appendix A Supplementary data

Supplementary material: Table S1 Single-factor experimental design and values of the responses for the triple-layered core-shell nanogel. Table S2 ANOVA of LC model. Table S3 ANOVA of EE model. Table S4 Blood biochemical assays of mice after oral administration of triple-layered core–shell nanogel for consecutive 14 d. Table S5 Blood physiological indexes of mice after oral administration of triple-layered core–shell nanogel for consecutive 14 d. Fig. S1: The antibacterial effect of the triple-layered core-shell nanogel against *S. aureus*.
