# Supplementary material for: Triple-layered nanogel for targeted and sustained delivery of florfenicol against bacterial enteritis
Source: Int J Pharm X. 2026 Aug 12;12:100637. doi: 10.1016/j.ijpx.2026.100637 (PMC13520975; doi:10.1016/j.ijpx.2026.100637)
Supplement: Supplementary file 1 [file mmc1.docx]

**Supplementary Materials for**

**Triple-Layered Nanogel for Targeted and Sustained Delivery of Florfenicol Against Bacterial Enteritis**

Tianhui Wang^1#^, Xingwang Ge^1#^, Yidan Zhang^2#^, Aizhi Han^3^, Samah Attia Algharib^4^, Keyu Zhang^5^, Muhammad Usman Ghani^2^, Shuo Han^1*^, Xiuge Gao^2*^, Wanhe Luo^1*^

^1^Engineering Laboratory for Tarim Animal Diseases Diagnosis and Control, College of Animal Science and Technology, Tarim University, Alar 843300, China

^2^Engineering Center of Innovative Veterinary Drugs, Center for Veterinary Drug Research and Evaluation, College of Veterinary Medicine, Nanjing Agricultural University, Nanjing 210095, China

^3^Instrumental Analysis Center, Tarim University, Alar 843300, China

^4^Department of Clinical Pathology, Faculty of Veterinary Medicine, Benha University, Moshtohor, Toukh 13736, Egypt

^5^Key Laboratory of Veterinary Drug Safety Evaluation and Residues Research, Shanghai Veterinary Research Institute, Chinese Academy of Agricultural Sciences, Shanghai 200241, China.

^#^Author contributions: Tianhui Wang, Xingwang Ge and Yidan Zhang contributed equally.

^*^Corresponding author: Shuo Han, E-mail: [15645977635@163.com](mailto:15645977635@163.com)

Xiuge Gao, E-mail: vetgao@njau.edu.cn

Wanhe Luo, E-mail: [luowanhe0728@163.com](mailto:luowanhe0728@163.com;)

**Data Availability**

The data employed to corroborate the findings of this study are available from the corresponding author upon request.

Table S1 Single-factor experimental design and values of the responses for the triple-layered core-shell nanogel.

| Std | CMCNa(mg/mL) | QCS(mg/mL) | TPP(mg/mL) | LC(%) | EE(%) |
| --- | --- | --- | --- | --- | --- |
| 1 | 30 | 10 | 10 | 15.6 | 50.3 |
| 2 | 90 | 10 | 10 | 16.4 | 64.7 |
| 3 | 30 | 100 | 10 | 17.3 | 73.2 |
| 4 | 90 | 100 | 10 | 22.6 | 82.7 |
| 5 | 30 | 10 | 40 | 16.9 | 51.6 |
| 6 | 90 | 10 | 40 | 17.3 | 72.8 |
| 7 | 30 | 100 | 40 | 17.4 | 72.7 |
| 8 | 90 | 100 | 40 | 20.5 | 81.6 |
| 9 | 30 | 50 | 20 | 20.4 | 76.4 |
| 10 | 90 | 50 | 20 | 22.2 | 86.0 |
| 11 | 60 | 10 | 20 | 19.0 | 70.1 |
| 12 | 60 | 100 | 20 | 21.3 | 84.6 |
| 13 | 60 | 50 | 10 | 22.4 | 83.6 |
| 14 | 60 | 50 | 40 | 22.8 | 84.4 |
| 15 | 60 | 50 | 20 | 23.1 | 87.6 |

Table S2 ANOVA of LC model.

| Source | Sum of Squares | df | Mean Square | F-value | P-value |  |
| --- | --- | --- | --- | --- | --- | --- |
| Model | 142.56 | 9 | 15.84 | 168.16 | <0.0001 | significant |
| A-CMCNa | 12.91 | 1 | 12.91 | 137.09 | <0.0001 |  |
| B-QCS | 18.31 | 1 | 18.31 | 194.40 | <0.0001 |  |
| C-TPP | 0.0240 | 1 | 0.0240 | 0.2544 | 0.6249 |  |
| AB | 6.59 | 1 | 6.59 | 69.98 | <0.0001 |  |
| AC | 0.7604 | 1 | 0.7604 | 8.07 | 0.0175 |  |
| BC | 2.02 | 1 | 2.02 | 21.44 | 0.0009 |  |
| A^2^ | 8.31 | 1 | 8.31 | 88.26 | <0.0001 |  |
| B^2^ | 25.63 | 1 | 25.63 | 272.04 | <0.0001 |  |
| C^2^ | 0.5982 | 1 | 0.5982 | 6.35 | 0.0304 |  |
| Residual | 0.9420 | 10 | 0.0942 |  |  |  |
| Lack of Fit | 0.9420 | 5 | 0.1884 |  |  |  |
| Pure Error | 0.0000 | 5 | 0.0000 |  |  |  |
| Cor Total | 143.51 | 19 |  |  |  |  |
| Std.Dev. | 0.3069 |  |  |  |  |  |
| Mean | 20.54 |  |  |  |  |  |
| C.V.% | 1.49 |  |  |  |  |  |
| R^2^ | 0.9934 |  |  |  |  |  |
| Adjusted R^2^ | 0.9875 |  |  |  |  |  |
| Predicted R^2^ | 0.9030 |  |  |  |  |  |
| Adeq Precision | 34.6732 |  |  |  |  |  |

Table S3 ANOVA of EE model.

| Source | Sum of Squares | df | Mean Square | F-value | P-value |  |
| --- | --- | --- | --- | --- | --- | --- |
| Model | 2531.21 | 9 | 281.25 | 134.20 | <0.0001 | significant |
| A-CMCNa | 403.74 | 1 | 403.74 | 192.66 | <0.0001 |  |
| B-QCS | 709.92 | 1 | 709.92 | 338.76 | <0.0001 |  |
| C-TPP | 6.93 | 1 | 6.93 | 3.31 | 0.0990 |  |
| AB | 35.64 | 1 | 35.64 | 17.01 | 0.0021 |  |
| AC | 6.63 | 1 | 6.63 | 3.16 | 0.1056 |  |
| BC | 12.25 | 1 | 12.25 | 5.85 | 0.0362 |  |
| A^2^ | 95.48 | 1 | 95.48 | 45.56 | <0.0001 |  |
| B^2^ | 315.32 | 1 | 315.32 | 150.46 | <0.0001 |  |
| C^2^ | 31.13 | 1 | 31.13 | 14.85 | 0.0032 |  |
| Residual | 20.96 | 10 | 2.10 |  |  |  |
| Lack of Fit | 20.96 | 5 | 4.19 |  |  |  |
| Pure Error | 0.0000 | 5 | 0.0000 |  |  |  |
| Cor Total | 2552.17 | 19 |  |  |  |  |
| Std.Dev. | 1.45 |  |  |  |  |  |
| Mean | 78.01 |  |  |  |  |  |
| C.V.% | 1.86 |  |  |  |  |  |
| R^2^ | 0.9918 |  |  |  |  |  |
| Adjusted R^2^ | 0.9844 |  |  |  |  |  |
| Predicted R^2^ | 0.8926 |  |  |  |  |  |
| Adeq Precision | 36.4117 |  |  |  |  |  |

Table S4 Blood biochemical assays of mice after oral administration of triple-layered core–shell nanogel for consecutive 14 d (n=3).

| Group | Unit | Control | Nanogel |
| --- | --- | --- | --- |
| TP | g/L | 50.53±0.26 | 50.95±1.05 |
| ALB | g/L | 30.89±0.61 | 30.36±0.32 |
| GLU | mmol/L | 15.52±0.21 | 15.06±0.28 |
| ALT | U/L | 29.08±1.05 | 28.97±2.12 |
| AST | U/L | 59.43±3.11 | 58.81±2.32 |
| TG | mmol/L | 1.41±0.17 | 1.50±0.02 |
| CREA | μmol/L | 25.85±0.42 | 24.79±0.05 |
| UREA | mmol/L | 5.52±0.31 | 5.65±0.06 |

Table S5 Blood physiological indexes of mice after oral administration of triple-layered core–shell nanogel for consecutive 14 d (n=3)

| Group | Unit | Control | Nanogel |
| --- | --- | --- | --- |
| WBC | 10^9^/L | 4.42±0.15 | 4.35±0.10 |
| Lym# | 10^9^/L | 2.52±0.79 | 2.48±1.62 |
| Mon# | 10^9^/L | 0.07±0.02 | 0.08±0.02 |
| Neu# | 10^9^/L | 0.78±0.12 | 0.75±0.13 |
| Lym% | % | 51.49±0.54 | 52.10±0.83 |
| Mon% | % | 1.38±0.33 | 1.42±0.24 |
| Neu% | % | 16.89±0.27 | 17.09±0.63 |
| RBC | 10^12^/L | 6.62±0.52 | 6.75±0.35 |
| HGB | g/L | 126.55±12.06 | 127.59±10.52 |
| HCT | % | 34.05±0.78 | 33.59±0.47 |
| MCV | fL | 56.49±0.67 | 55.92±0.58 |
| MCH | pg | 21.84±0.85 | 22.81±0.34 |
| MCHC | g/L | 364.84±2.51 | 361.73±1.24 |
| RDW | % | 15.47±0.75 | 14.95±0.39 |
| PLT | 10^9^/L | 548.83±11.55 | 546.86±12.42 |
| MPV | fL | 4.73±0.26 | 4.82±0.43 |
| PDW | % | 15.09±0.32 | 15.31±0.34 |
| PCT | % | 0.32±0.01 | 0.38±0.02 |

Fig. S1: The antibacterial effect of the triple-layered core-shell nanogel against *S. aureus*. MICs (A); Inhibition zones (B); *In vitro* killing curves (C); Colony counting (D); SEM image (E).
